# Expression Pattern and Functional Analyses of *Arabidopsis* Guard Cell-Enriched GDSL Lipases

**DOI:** 10.3389/fpls.2021.748543

**Published:** 2021-09-21

**Authors:** Chuanlei Xiao, Huimin Guo, Jing Tang, Jiaying Li, Xuan Yao, Honghong Hu

**Affiliations:** ^1^National Key Laboratory of Crop Genetic Improvement, College of Life Science and Technology, Huazhong Agricultural University, Wuhan, China; ^2^College of Plant Science and Technology, Huazhong Agricultural University, Wuhan, China

**Keywords:** Arabidopsis, drought tolerance, expression pattern, guard cells, GDSL lipases, stomatal density, stomatal dynamics, subcellular localization

## Abstract

There are more than 100 GDSL lipases in *Arabidopsis*, but only a few members have been functionally investigated. Moreover, no reports have ever given a comprehensive analysis of GDSLs in stomatal biology. Here, we systematically investigated the expression patterns of 19 putative *Guard-cell-enriched GDSL Lipases* (*GGLs*) at various developmental stages and in response to hormone and abiotic stress treatments. Gene expression analyses showed that these *GGLs* had diverse expression patterns. Fifteen *GGLs* were highly expressed in guard cells, with seven preferentially in guard cells. Most GGLs were localized in endoplasmic reticulum, and some were also localized in lipid droplets and nucleus. Some closely homologous *GGLs* exhibited similar expression patterns at various tissues and in response to hormone and abiotic stresses, or similar subcellular localization, suggesting the correlation of expression pattern and biological function, and the functional redundancy of *GGLs* in plant development and environmental adaptations. Further phenotypic identification of *ggl* mutants revealed that *GGL7*, *GGL14*, *GGL22*, and *GGL26* played unique and redundant roles in stomatal dynamics, stomatal density and morphology, and plant water relation. The present study provides unique resources for functional insights into these GGLs to control stomatal dynamics and development, plant growth, and adaptation to the environment.

## Introduction

GDSL lipases or esterases (EC 3.1.1.3) are lipid hydrolases with a GDSL motif at the N-terminus. GDSLs have four invariant important catalytic residues: Ser, Gly, Asn, and His in blocks I, II, III, and V, respectively (Akoh et al., 2004). GDSLs widely exist in prokaryotes and eukaryotes. In plants, it exists as a big family with many members, more than 100 members in *Arabidopsis* (Ling, 2008; Dong et al., 2016; Lai et al., 2017; Su et al., 2020), 114 members in rice (Chepyshko et al., 2012), 121 in *Brassica rapa* (Dong et al., 2016), and 194 in soybean (Su et al., 2020). However, only a few members have been identified in each plant species with their broad biological functions and substrates.

GDSLs play roles in plant growth and organ development. *Arabidopsis* EXL4 (EXTRACELLULAR LIPASE 4) is required for pollen on stigma to hydrate efficiently. Loss function of *EXL4* led to the delayed and reduced rate of pollen hydration (Mayfield et al., 2001; Updegraff et al., 2009). CDEF1 (CUTICLE DESTRUCTING FACTOR 1) acts as a cutinase, which directly degrades the polyester in the cuticle of stigma and mediates pollen tube penetration into the stigma (Takahashi et al., 2010). Tomato GDSL1 plays a specific role in cutin polyester deposition in the tomato fruit cuticle (Girard et al., 2012), and CD1 is required for cutin accumulation by catalyzing 2-MHG *in vivo* and catalyzes the formation of primarily linear cutin oligomers *in vitro* (Yeats et al., 2012, 2014). Two rice GDSLs, BS1 (Brittle Leaf Sheath 1) and DARX1 (DEACETYLASE ON ARABINOSYL SIDECHAIN OF XYLAN 1), are identified as deacetylases that are crucial for secondary wall formation and patterning. BS1 cleaves acetyl moieties from xylopyranosyl residues (Zhang et al., 2017a), and DARX1 specifically deacetylates the side chain of the major rice hemicellulose, arabinoxylan (Zhang et al., 2019). *ZmMs30*, a maize genic male sterility gene, regulates male fertility by modulating cuticle deposition on anthers (An et al., 2019). OsGELP34, OsGELP110, and OsGELP115 control male fertility by regulating exine formation (Zhang et al., 2020). BnSCE3 serves as a sinapine esterase that controls seed weight, size, and water content (Ling et al., 2006; Clauss et al., 2008, 2011).

GDSLs regulate plant adaptation to biotic and abiotic stresses. *Arabidopsis* GDSL LIPASE1 (GLIP1) is a critical component in plant resistance to several bacterial and fungal pathogens, directly disrupting fungal spore integrity and inhibiting its germination (Oh et al., 2005; Kwon et al., 2009). Pepper GLIP1 plays as a negative regulator in resistance to *Xanthomonas campestris pv. vesicatoria* (Xcv) (Hong et al., 2008). Rice GLIP1 and GLIP2 act as negative regulators of disease resistance to bacterial and fungal pathogens by changing the levels of DGDG and MGDG (Gao et al., 2017). *Arabidopsis* Li-tolerant lipase 1 (AtLTL1) increases salt tolerance of *Arabidopsis* and LiCl tolerance of yeast (Naranjo et al., 2006). Rice WDL1 (Wilted Dwarf and Lethal 1) mediates water loss by regulating wax synthesis (Park et al., 2010). Our recent research has shown that *Arabidopsis* OSP1 (Occlusion of Stomatal Pore 1) confers drought tolerance through the control of wax biosynthesis, stomatal outer cuticular ledge formation, and stomatal density (Tang et al., 2020). However, the functions of most GDSLs are unexplored.

GDSL lipase has a flexible active site (Akoh et al., 2004), which leads to catalytic activity on different substrates by changing conformations. Due to this changeable structure feature, isolation and characterization of GDSL substrates is a big challenge. For example, a bread wheat (*Triticum aestivum*) xanthophyll acyltransferase (XAT) has broad substrate specificity. XAT can esterify lutein, β-cryptoxanthin, and zeaxanthin (Watkins et al., 2019). Tremendously functional redundancy and tandem duplications in chromosomes could be other challenges to identify the biological functions of GDSLs (Lai et al., 2017). Their functions may only be determined when higher-order mutants are generated by crossing, CRISPR/Cas9 gene editing, or artificial microRNA technologies (Feng et al., 2013; Hauser et al., 2013; Miao et al., 2013). Therefore, detailed expression patterns are critical for characterizing the functions of GDSL lipases in plant development, plant growth, and adaptation to the environment.

Stomata are pores formed by pairs of guard cells in the surface of aerial parts of most higher plants, which respond quickly to the environmental changes by opening or closing the pores. It has been suggested that manipulation of stomatal development and behavior is a good strategy for improving plant abiotic and biotic tolerance (Hughes et al., 2017; Dunn et al., 2019; Papanatsiou et al., 2019; Huang et al., 2021). GDSLs exist as a big family, but only OSP1 has been identified with essential roles in stomata (Tang et al., 2020). Therefore, it is very important to identify GDSLs that function in stomata. In this study, we identified 29 predicted *GGLs* (*Guard-cell-enriched GDSL Lipases*) from microarray data and determined the temporal–spatial expression patterns of 19 *GGLs* by driving the *GUS* reporter gene in *Arabidopsis*. We also explored their cellular localizations by transient expression of GFP or YFP fused GGLs in *Nicotiana benthamiana*. Furthermore, we investigated the roles of six guard cell preferentially expressed *GGLs* in stomatal biology and plant water maintenance. Our data provide unique resources for the future investigation of the roles of *GGLs* in controlling stomatal dynamics and stomatal development, plant growth, and adaptation to the environment.

## Materials and Methods

### Plant Materials and Growth Conditions

*Arabidopsis* (*Arabidopsis thaliana*) accession Col-0 and *N. benthamiana* plants were used in this study. The single T-DNA insertion mutants, *ggl7* (CS393512), *ggl12* (SALK_024323C), *ggl14* (SALK_106116C), *ggl22* (SALK_062226C), *ggl23* (CS874407), *ggl26* (SALK_116756), and *ggl27* (CS857064), were obtained from the Arabidopsis Biological Resource Center (ABRC). The *Arabidopsis* plants and *N. benthamiana* were grown in a well-controlled growth chamber or a greenhouse at 22°C with a 16h light/8h dark regime.

### Plasmid Construction

To generate the promoter::*GUS* expression vectors, we cloned 1.5–2kb promoter regions (DNA fragment upstream of the ATG start codon) into the expression vector *pLP100* or *pMDC163* (Szabados et al., 1995; Charrier et al., 1996; Curtis and Grossniklaus, 2003). All promoter sequences were confirmed by DNA sequencing, and the primers used are listed in Supplementary Table S2.

To investigate the subcellular localization of the predicted GGLs, we amplified the open reading frames of GGLs from cDNA of Col-0 seedlings using gene-specific primers (Supplementary Table S2). PCR products were cloned into the Gateway-compatible donor vector *pDONR207* by BP recombination reactions to generate entry clones and confirmed by DNA sequencing. Subsequently, the positive entry clones were further cloned into the destination vector *pGWB541* or *BarII-pUBQ10-GWB-GFP* (Walter et al., 2004; Nakagawa et al., 2007) by LR recombination reactions.

### Generation of Transgenic Plants and GUS Staining

The generated *GGL*pro::*GUS* constructs were transformed into Col-0 plants by flower dipping method (Zhang et al., 2006). Transgenic plants were screened by Kanamycin or Hygromycin B. Positive transgenic plants were further confirmed by detecting the existence of the *GUS* reporter gene. The transgenic seedlings of 1.5days after germination (DAG), 6 DAG, and 14 DAG growing on 1/2 Murashige and Skoog medium supplemented with 1% sucrose and 0.3% phytagel were used for GUS staining. The representative lines showing consistent GUS staining were further analyzed for GUS staining at the reproductive stage (34 DAG). At least three independent transgenic lines were analyzed in parallel.

The seedlings or tissues were immersed in GUS solution buffer [1mg/ml X-Gluc, 5mM K_3_Fe(CN)_6_, 5mM K_4_Fe(CN)_6_·3H_2_O, 0.042M NaH_2_PO_4_·2H_2_O, 0.058M Na_2_HPO_4_·12H_2_O, 0.1mM Na_2_EDTA (pH=8.0), and 1% (v/v) Triton X-100], and incubated overnight at 37°C. After staining, the seedlings and tissues were de-stained in 75% ethanol several times for GUS observation under a microscope.

### Subcellular Localization

The constructs of *UBQ10-GGL-GFP* or *35S-GGL-YFP* were transformed into the *Agrobacterium* strain GV3101, and the strains were infiltrated into *N. benthamiana* leaf epidermis. Protoplasts of infiltrated tobacco leaves were prepared as described previously (Walter et al., 2004). Images were obtained by a confocal microscope (TCS-SP8; Leica, Weztlar, Germany) with a 40× water-immersion objective in the sequential scan, between frames mode. For localization in ER, an ER-marker HDEL-OFP (orange fluorescent protein; excitation at 561nm, emission range is 580nm to 630nm) was coexpressed for co-localization. Nile Red staining was performed for localization in lipid droplets, as described in our previous publication (Tang et al., 2020).

To confirm the subcellular localization of GGL13, GGL17, and GGL27 in *Arabidopsis*, GGL13-GFP, GGL17-GFP, or GGL27-GFP was transformed into *Arabidopsis* mesophyll protoplasts with HDEL-OFP (Yoo et al., 2007), respectively. The GFP and OFP signals of protoplasts were recorded 10–12h after transformation under a confocal microscope (TCS-SP8; Leica, Weztlar, Germany).

### Transpiration Rate, Water Use Efficiency, and Stomatal Conductance Analyses

Transpiration rate was determined on rosette leaves of 4-week-old plants using a portable photosynthesis system (LI-6400XT; Li-Cor). The measurement conditions were 150μmolm^−2^ s^−1^ light intensity, 50–60% relative humidity, and 450ppm CO_2_. Measurements were recorded every 30s and lasted for 20min. Data presented are the average value of 10min for individual plants (at least four plants per genotype) for each experiment. Instantaneous water use efficiency (WUE) was defined as the ratio of CO_2_ assimilated to water loss during transpiration (μmol CO_2_ mmol H_2_O^−1^). WUE was calculated using the data collected during transpiration rate measurement. The corresponding time points (10min) were chosen for each plant. Experiments were repeated at least three times.

For stomatal conductance in response to dark-to-light (150μmolm^−2^ s^−1^ with 10% blue light) transitions, intact leaves of 4 to 5-week-old well-growing plants were measured by a portable gas exchange analyzer (LI-6400XT; Li-Cor). According to the previous publication (Hu et al., 2010), the initial rate of stomatal conductance changes in response to dark-to-light transitions was calculated.

### Stomatal Density and Stomatal Morphology Analyses

The seventh or eighth (including cotyledons) rosette leaves of 4-week-old plants were analyzed for stomatal density and index, stomatal pore width and pore ratio (width: length), and stomatal complex length and width. All plants were grown in a well-controlled growth room at 22°C, with 56% humidity and a 16h light/8h dark photoperiod regime with 80μmolm^−2^ s^−1^ light intensity. The central areas derived from the leaf abaxial epidermal layer were imaged using a light microscope (TS100, Nikon, Japan). Stomata and pavement cell numbers were counted with ImageJ software. Stomatal pore width and length, and stomatal complex length and width were measured with ImageJ software. Experiments were repeated three times.

### Stress Treatment, RT-PCR, and Real-Time Quantitative PCR Analyses

For different hormone treatments, 7-day-old seedlings growing on 1/2 MS plates were treated with different phytohormones, including 10μM ABA (abscisic acid), 10nM BL (brassinolide), 1μM GA (gibberellin), 1μM IAA (indoleacetic acid), and solvent (as control). The seedlings were harvested at 0, 0.5, 1, and 3h after treatments, respectively. For salt stress, 4-week-old plants were treated (watered) with 150mM NaCl, and the leaf samples were harvested at the time points of 0, 0.5, 1, 3, 6, 12, and 24h. For dehydration treatment, rosette leaves were detached from 4-week-old plants and dehydrated under the laboratory conditions. The samples were harvested at the time points of 0, 0.25, 0.5, 1, 3, 6, and 12h after leaf detachments. Total RNA was extracted from 50 to 100 mg of sample tissues using TRIZOL Reagent (Invitrogen). After DNase treatment, the first-strand cDNA was synthesized from 2μg of RNA using oligo (dT) primers with M-MLV reverse transcriptase (Promega). For RT-PCR analyses, 100ng cDNA was used as templates for amplification of *Actin7* and *GGLs*. 30–32cycles were amplified. Primers used for RT-PCR are listed in Supplementary Table S2.

Real-time quantitative PCR was performed with the Bio-Rad CFX96^™^ Real-Time System using SYBR (Vazyme) to monitor double-stranded DNA products. *EFα* was used as an internal control. The relative gene expression during different treatments was calculated by comparison with that of the samples at 0h, which was defined as 1. Bio-Rad CFX manager software was used for analysis. Primers used for real-time quantitative PCR are listed in Supplementary Table S2.

### Drought Stress Assay

Plants (each pot containing 25 plants with the same weight of soil and the same water content) were grown in well-watered conditions for 3weeks. Then, water was withdrawn for 8–10days until significant differences in the wilted leaves were observed and re-supplied for 2 days. Photographs of the plants at these three time points were taken.

## Results

### Identification of *GDSLs* Enriched in *Arabidopsis* Guard Cells

**Figure 1:**
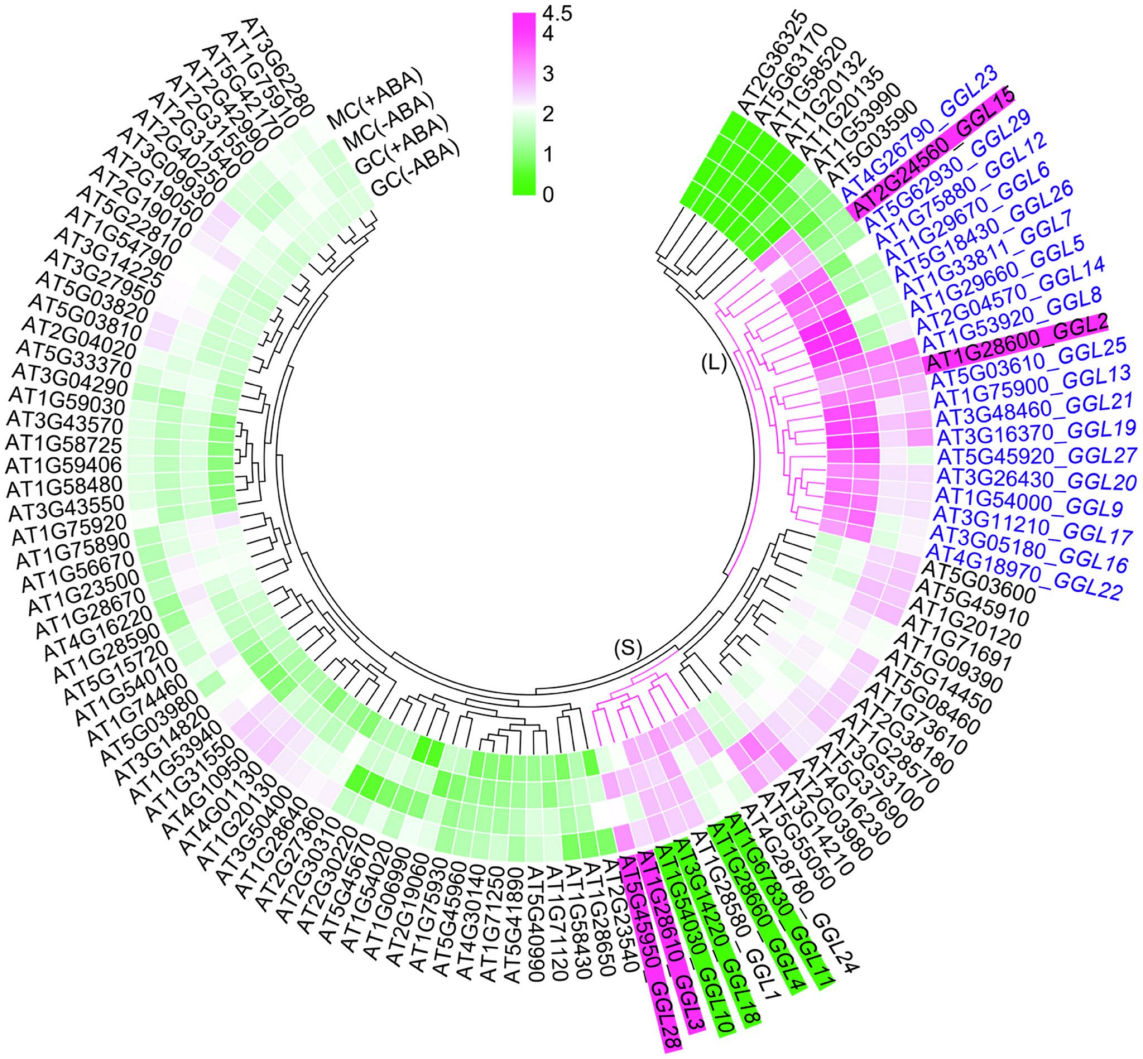
Identification of *GGLs* in *Arabidopsis*. Expression levels of *Arabidopsis GDSLs* in guard cell and mesophyll cell protoplasts in response to ABA or not. ATH1 microarray data of all *Arabidopsis GDSLs* in guard cell (GC) and mesophyll cell (MC) protoplasts treated with ABA or not were obtained from Leonhardt et al. (2004) and Yang et al. (2008). Putative *GDSLs* were merged from Dong et al. (2016) and Lai et al. (2017). The heat map was constructed by TBtools (Chen et al., 2020), and the color code of signal intensities corresponds to the abundance of transcripts, from low (green) to high (magenta) expression. *GGL* means *Guard-cell-enriched GDSL Lipase*. (L) and (S) indicate the large and small clades, respectively. Nineteen *GGLs* in L clade were shown in blue fonts. The *GGLs* highlighted in magenta or green indicated the *GGLs* in guard cell protoplasts induced or repressed by ABA treatment, respectively.

To gain insights into the GDSLs that function in stomatal biology, we focused on the guard cell highly expressed *GDSLs* in the *Arabidopsis* genome. Firstly, we extracted the expression data of all putative *GDSL* genes from the microarray data of guard cell and mesophyll cell protoplasts with or without ABA treatment published by Leonhardt et al. (2004) and drew a heat map with TBtools (Chen et al., 2020). The results showed that 29 *GDSLs* belonging to a large clade (L) and a small one (S) had relatively higher expression levels in guard cell protoplasts (Figure 1). We then named these *GDSLs* as *GGLs* (*Guard-cell-enriched GDSL Lipases*). Among these 29 *GGLs*, the expression levels of *GGL2* (AT1G28600), *GGL3* (AT1G28610), *GGL15* (AT2G24560), and *GGL28* (AT5G45950) in guard cell protoplasts were upregulated by ABA treatment, while those of another four *GGLs*, *GGL4* (AT1G28660), *GGL10* (AT1G54030), *GGL11* (AT1G67830), and *GGL18* (AT3G14220), were slightly repressed by ABA treatment (Figure 1). The remaining *GGLs* were not affected by ABA treatment in guard cell protoplasts (Figure 1). Moreover, the expression levels of *GGLs* in L clade were generally higher than those in S clade, and 19 *GGLs* from the L clade were preferentially expressed in guard cells than those in mesophyll cells (Figure 1).

We then analyzed the distribution of these *GGLs* on chromosomes by Chromosome Map Tool.^1^ These *GGLs* were distributed on all chromosomes. Thirteen *GGLs* were located on chromosome 1, 6 on chromosome 3, 3 on chromosome 4, and 5 on chromosome 5, whereas only two were located on chromosome 2 (Supplementary Figure S1). Furthermore, there were cases of two or more *GGLs* arranged in tandem, on the middle and bottom of chromosome 1 (Supplementary Figure S1). For example, *GGL1*, *GGL2*, and *GGL3* were tandem duplicated (Supplementary Figure S1). Given that tandem repeated genes often show functional redundancy (Tantikanjana et al., 2004; Su et al., 2013), we speculate that tandem repeated *GGLs* might have functional redundancy.

### Tissue-Specific Expression Patterns of *GGLs* at the Seedling Stage

**Figure 2:**
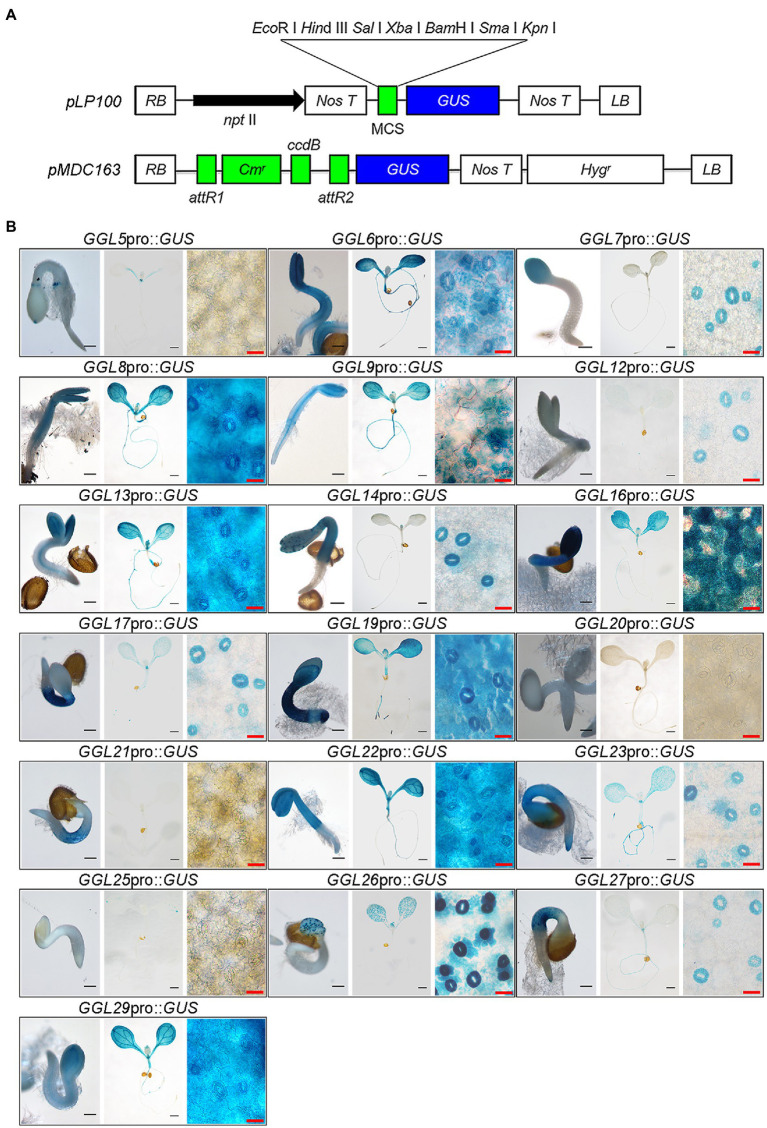
Expression patterns of 19 *GGLs* at the early seedling stage. (**A**) Schematic charts of two destination vectors (*pLP100* and *pMDC163*) used for *GUS* expression driven by *GGL* native promoters. (**B**) Expression profile analyses of 19 *GGLs* at the early seedling stage. *GGL*pro::*GUS* expressing transgenic seedlings that grown in a growth chamber were stained with X-Gluc. For each gene, the images from left to right represent a seedling of 1.5days after germination (DAG; scale bar=200μm), a seedling of 6 DAG (scale bar=1mm), and an enlarged part of the cotyledon from the 6-DAG seedling (scale bar=25μm), respectively.

To confirm that these predicted *GGLs* in L clade are highly expressed in guard cells, we cloned the regions of 1.5–2.0kb of DNA fragments upstream of the start codon (ATG) for these 19 *GGLs* (marked in blue fonts in Figure 1) as native promoters into the expression vector *pLP100* or *pMDC163* to drive the expression of *GUS* reporter gene (Figure 2A). These 1.5–2.0kb regions should have contained enough regulatory elements to drive the expression of most *Arabidopsis* genes (Korkuæ et al., 2014; Wu et al., 2016). All these constructs were transformed into the wild-type *Arabidopsis* Col-0 accession.

We performed GUS staining of transgenic plants expressing *GGL*pro::*GUS* in different tissues at different developmental stages. At least three independent transgenic lines were used for analyses, and only those lines showing the most consistent patterns were photographed. At 1.5 DAG (Days After Germination), 16 *GGLs* were highly expressed in the emerged cotyledons or hypocotyls, whereas *GGL12*, *GGL20*, and *GGL25* had very weak expressions (Figure 2B). Seventeen from 19 *GGLs*, except *GGL20* and *GGL21*, were expressed in the 6-DAG seedlings (Figure 2B). Ten members (*GGL6*, *GGL8*, *GGL9*, *GGL13*, *GGL16*, *GGL19*, *GGL22*, *GGL23*, *GGL26*, and *GGL29*) were highly expressed in cotyledons, and nine members (*GGL6*, *GGL8*, *GGL9*, *GGL13*, *GGL16*, *GGL19*, *GGL22*, *GGL23*, and *GGL27*) showed evident expressions in roots (Figure 2B). Interestingly, 7 *GGLs* (*GGL7*, *GGL12*, *GGL14*, *GGL17*, *GGL23*, *GGL26*, and *GGL27*) were preferentially expressed in guard cells (Figure 2B), indicating that they may function in stomata. Eight *GGLs* (*GGL6*, *GGL8*, *GGL9*, *GGL13*, *GGL16*, *GGL19*, *GGL22*, and *GGL29*) were expressed not only in cotyledon guard cells but also in pavement or mesophyll cells (Figure 2B), suggesting their potential roles in other physiological processes in addition to stomatal biology.

**Figure 3:**
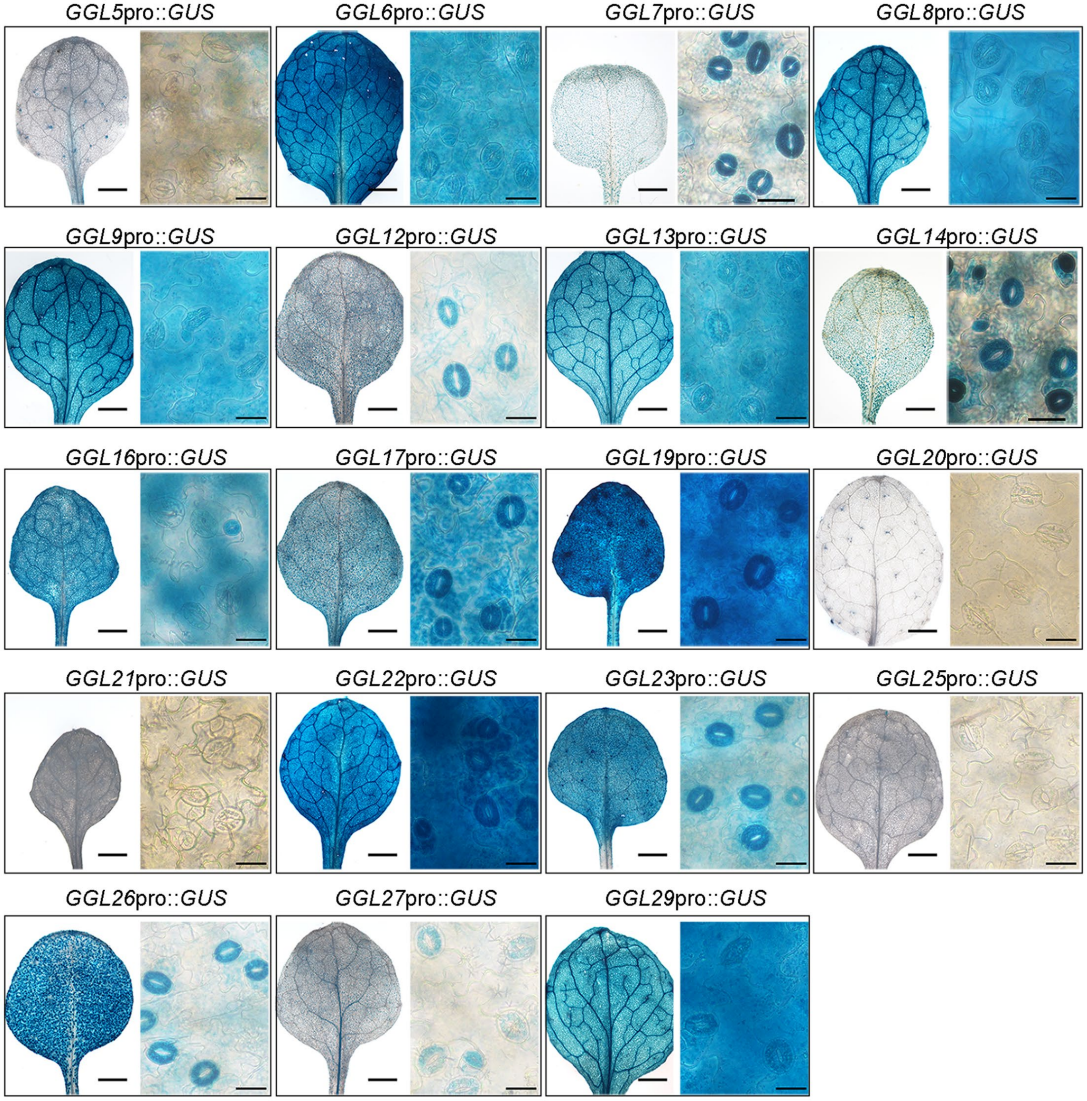
Expression profiles of 19 *GGLs* in 14-DAG true leaves. The true leaves of 14-DAG *GGL*pro::*GUS* expressing seedlings were stained with X-Gluc. For each gene, the images from left to right represent a true leaf from a 14-DAG seedling (scale bar=1mm) and an enlarged part of it (scale bar=25μm).

To confirm that these *GGLs* are expressed in the guard cells of true leaves, we further determined their expression patterns in the true leaves of 14-DAG seedlings. Consistent with their expression patterns in cotyledons (Figure 2B), the same 15 *GGLs* were expressed in the guard cells of true leaves (Figure 3). Seven *GGLs* (*GGL7*, *GGL12*, *GGL14*, *GGL17*, *GGL23*, *GGL26*, and *GGL27*) were preferentially expressed in the true leaf guard cells, and eight members (*GGL6*, *GGL8*, *GGL9*, *GGL13*, *GGL16*, *GGL19*, *GGL22*, and *GGL29*) also showed evident expressions in pavement or mesophyll cells in addition to guard cells (Figure 3). Moreover, five *GGLs* (*GGL5*, *GGL14*, *GGL17*, *GGL19*, and *GGL23*) were also expressed in trichomes (Figure 3 and Supplementary Figure S2), and seven *GGLs* (*GGL6*, *GGL8*, *GGL9*, *GGL13*, *GGL16*, *GGL22*, and *GGL29*) were expressed in the vascular tissues (Figure 3), indicating that these GGLs may also be involved in trichome and vascular tissue development.

### Tissue-Specific Expression Patterns of *GGLs* at the Reproductive Tissues

**Figure 4:**
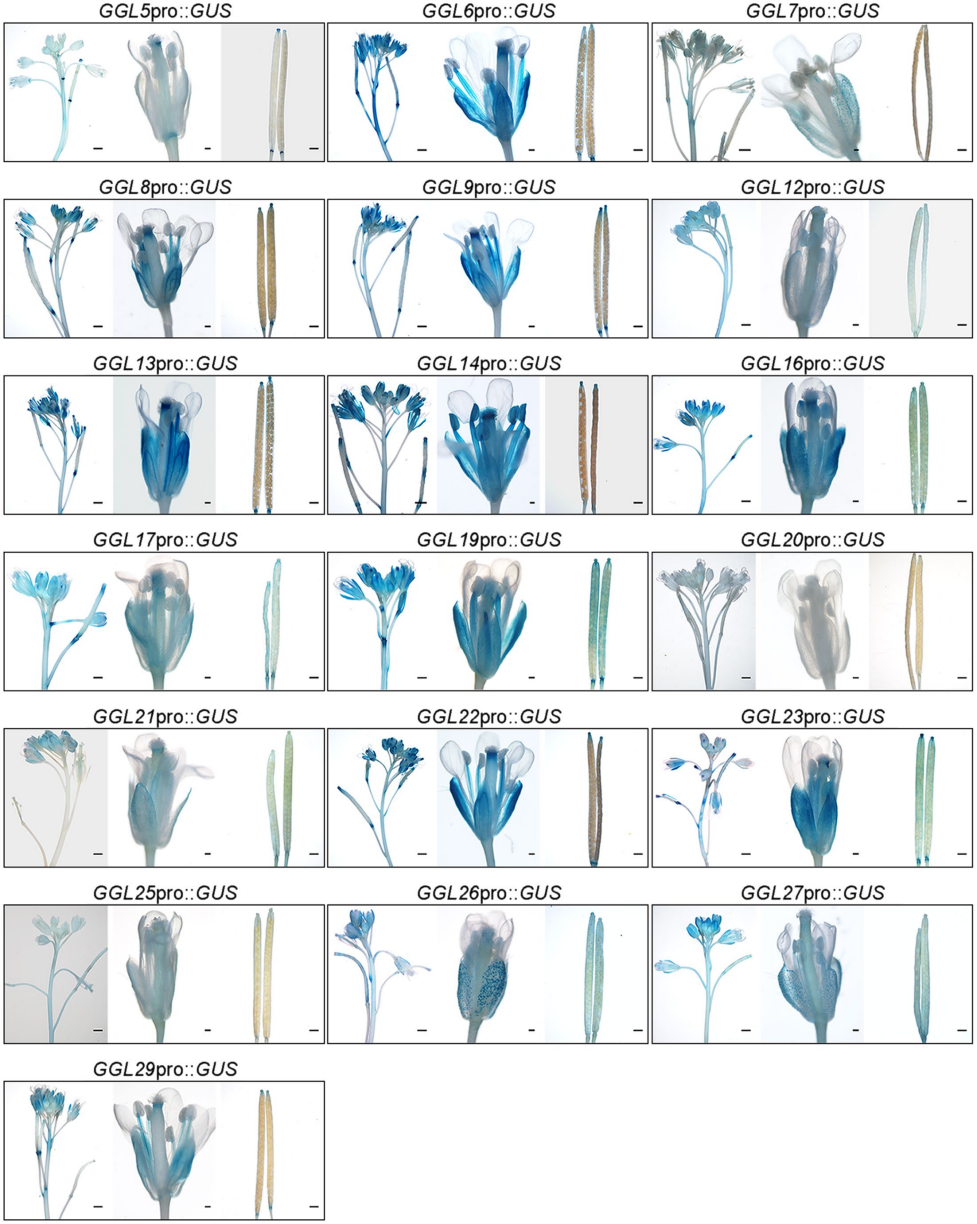
Expression patterns of 19 *GGLs* at reproductive stage. The tissues from 34-DAG *GGL*pro::*GUS* expressing plants were stained with X-Gluc. For each gene, the images from left to right represent an inflorescence from a 34-DAG plant (scale bar=100μm), a mature flower from a 34-DAG plant (scale bar=1mm), and mature siliques from a 34-DAG plant (scale bar=100μm), respectively.

We next determined the expression patterns of these *GGLs* at the reproductive stage. Among 19 *GGLs*, 18 (except *GGL20*) were expressed in the inflorescence of 34-DAG *Arabidopsis* plants (Figure 4). *GGL7*, *GGL26*, and *GGL27* were preferentially expressed in guard cells on sepals (Figure 4). *GGL6*, *GGL8*, *GGL9*, *GGL13*, *GGL14*, *GGL22*, and *GGL29* showed very similar expression patterns, with strong expressions in filaments, sepals, and apex of stigma (Figure 4), suggesting that these GGLs may be involved in flower development or fertility. The remaining *GGLs* had relatively narrow expression patterns. *GGL5* was expressed in the apex and base of stigma, *GGL16* and *GGL17* were expressed in sepals and apex of stigma, and *GGL19* was expressed in filaments and sepals (Figure 4). Moreover, *GGL14* and *GGL16* were also expressed in anthers. We also found that seven *GGLs* (*GGL12*, *GGL16*, *GGL17*, *GGL19*, *GGL23*, *GGL26*, and *GGL27*) were expressed in the whole siliques, and eight *GGLs* (*GGL5*, *GGL6*, *GGL8*, *GGL9*, *GGL13*, *GGL14*, *GGL22*, and *GGL29*) were expressed only in both ends of siliques (Figure 4).

### Subcellular Localization Analyses of *GGLs* in *N. benthamiana*

**Figure 5:**
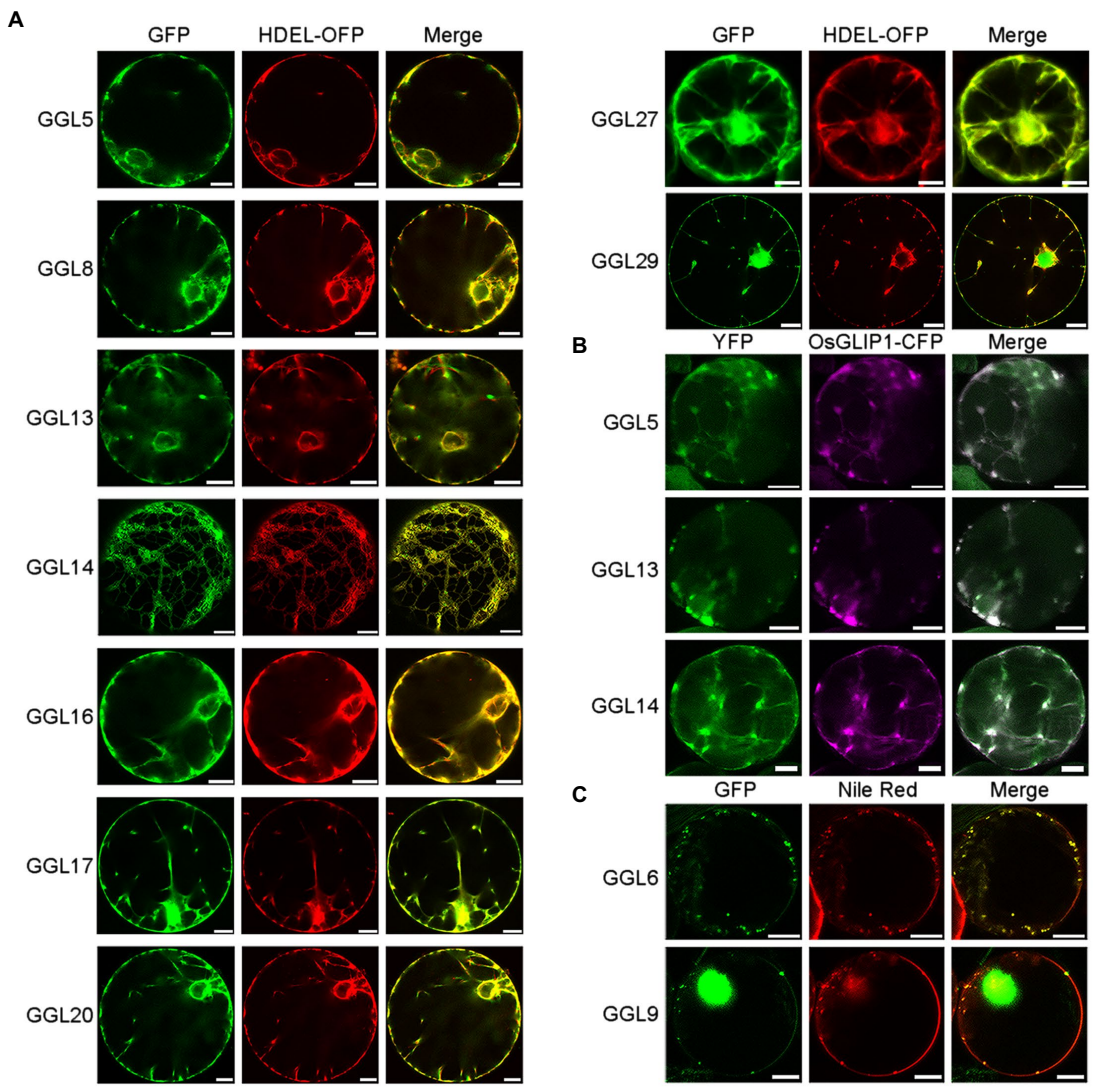
Subcellular localization of GGLs in protoplasts of *Nicotiana benthamiana* leaves. Subcellular localization of C-terminal GFP or YFP fused GGL proteins in *N. benthamiana* protoplasts (**A-C**). C-terminal GFP or YFP fused GGL proteins under the control of the *CaMV 35S* promoter were transiently expressed in *N. benthamiana* leaf epidermal cells. GFP or YFP signals in the isolated protoplasts were imaged by a confocal microscope. HDEL-OFP was coexpressed with GGLs to indicate endoplasmic reticulum localization (**A**). Lipid droplet localization of GGLs was confirmed by co-localization with OsGLIP1-CFP (Gao et al., 2017) (**B**) or Nile Red staining (**C**). Scale bar=10μm.

Several GDSLs have been reported to be secreted into the intercellular space; signal peptide prediction using SignalP 4.1 Server^2^ revealed that 14 of 19 GGLs possessed a signal peptide at N-terminus (Supplementary Table S1). To gain insights into which organelles GGLs are localized in plant cells, we investigated the subcellular localization of 13 GGLs tagged by GFP or YFP under the control of the cauliflower mosaic virus (*CaMV*) *35S* promoter by transient expression in *N. benthamiana* leaf epidermis, a convenient system to study protein intracellular localization (Deeks et al., 2012). Our results showed that most C-terminal GFP- or YFP-tagged GGL proteins were co-localized, at least partially, with the endoplasmic reticulum (ER) marker HDEL-OFP (Supplementary Figure S3A). To further confirm that these GGLs are localized in ER, we observed their localizations in the isolated protoplasts. Nine of thirteen GGLs (GGL5, GGL8, GGL13, GGL14, GGL16, GGL17, GGL20, GGL27, and GGL29) were well overlapped with HDEL-OFP (Figure 5A), demonstrating that these GGLs are localized in ER. Among nine ER-localized GGLs, three GGLs (GGL5, GGL13, and GGL14) also appeared as punctate localization in the cytoplasm (Figure 5A). We speculated that these vesicle structures were lipid droplets, and GGL5 and GGL13 could be dual localization proteins in both ER and lipid droplets as GGL14 (also named OSP1) did (Tang et al., 2020). Therefore, we performed co-localization of GGL5, GGL13, or GGL14 with OsGLIP1-CFP, a protein reported to localize in lipid droplets and ER (Gao et al., 2017), respectively. GGL5, GGL13, and GGL14 overlapped with OsGLIP1-CFP in the vesicle structures and ER networks (Figure 5B), suggesting that GGL5, GGL13, and GGL14 may also play roles in lipid homeostasis. GGL6 and GGL9 appeared in the vesicle structures in tobacco epidermal cells (Supplementary Figure S3B), and lipophilic Nile Red staining showed that these vesicle structures were lipid droplets (Figure 5C). Moreover, GGL9, GGL17, GGL27, and GGL29 were also localized in nucleus (Figures 5A,C and Supplementary Figure S3). We further validated the subcellular localization of three GGLs in *Arabidopsis* mesophyll protoplasts. The results showed that GGL13, GGL17, and GGL27 overlapped well with HDEL-OFP (Supplementary Figure S3C), consistent with their localizations in *N. benthamiana* leaf epidermis (Figure 5A). These results suggest that subcellular localization of these *Arabidopsis* GGLs in *N. benthamiana* leaf epidermis by our system is suitable and reliable.

### Phylogenetic Relationship and Exon-Intron Structures of *GGLs* in *Arabidopsis*

To investigate the evolutionary relationship between these 19 GGL proteins, we constructed a maximum likelihood tree using GGL protein sequences (Supplementary Figure S4). GGL5 showed a close relationship with GGL6, GGL7, and GGL22 (Supplementary Figure S4). GGL14 and GGL23, GGL12 and GGL13, and GGL16 and GGL20 were highly homologous proteins, respectively (Supplementary Figure S4). GGL17 exhibited a close relationship with GGL27 and GGL29 (Supplementary Figure S4). Some closely homologous GGLs, such as GGL5 and GGL6, and GGL12 and GGL13, were found to be arranged in tandem on chromosomes (Supplementary Figure S1). These closely related GGLs are mostly expressed in guard cells, indicating that they may function redundantly in stomatal biology.

We next analyzed the exon and intron structures of these 19 *GGLs* based on exon assignment information from the TAIR Web site.^3^ Among these *GGLs*, only *GGL12*, *GGL22*, and *GGL23* have two transcripts, and the others all have only one transcript (Supplementary Figure S4). Most *GGLs* contain five exons. *GGL14*, *GGL21*, and *GGL23* have three exons, and *GGL9* has four exons, whereas *GGL7*, *GGL17*, and *GGL22.2* have six exons (Supplementary Figure S4). We surprisingly found that *GGL20* was a unique one, which possessed a long 5′ untranslated region of about 2.5kb (Supplementary Figure S4), which may have a regulatory effect on its expression (Broad et al., 2019; Nitschke et al., 2020).

### Some *GGLs* Play Roles in Water Transpiration and Light-Induced Stomatal Opening

**Figure 6:**
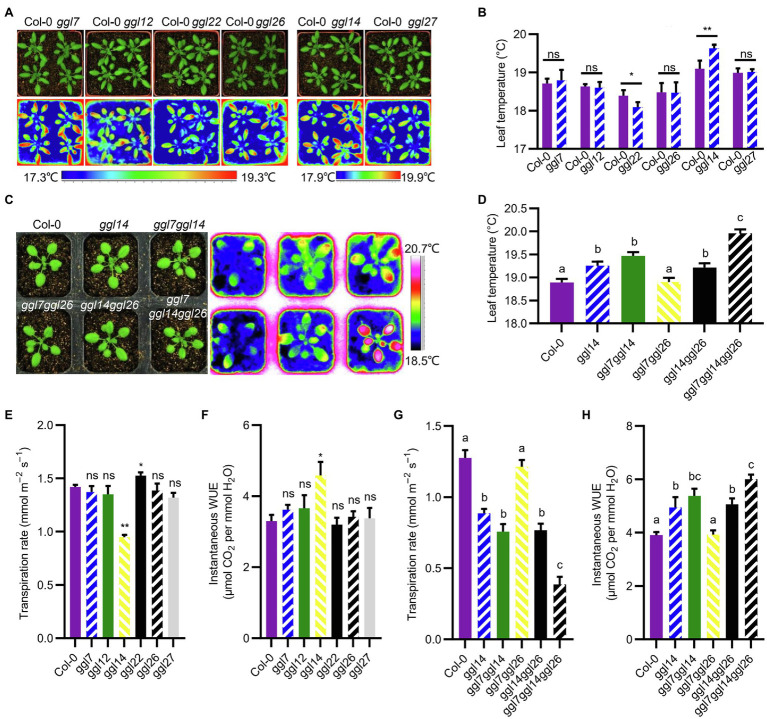
Leaf temperature, transpiration rate, and instantaneous water use efficiency (WUE) of *ggl* mutants. (**A**) Photograph (up) and infrared thermal imaging (down) of Col-0 and *ggl* single mutants. (**B**) Quantification and statistical analyses of leaf temperature of Col-0 and *ggl* single mutant plants in (**A**). Values are means ± SE (*n*=three independent experiments, each with four plants per genotype). ^*^p<0.05; ^**^p<0.01; ns, no significant difference; Student’s *t*-test. (**C**) Photograph (left) and infrared thermal imaging (right) of Col-0, *ggl14*, the corresponding double mutant, and triple mutant plants. (**D**) Quantification and statistical analyses of leaf temperature of Col-0, *ggl14*, the corresponding double mutant, and triple mutant plants in (**C**). Values are means ± SE (*n*=three independent experiments, each with six plants per genotype). Different letters indicate statistically significant differences (*p*<0.05) by one-way ANOVA and Tukey’s test analyses. (**E–H**) The transpiration rate and WUE of *ggl* mutants were measured using a portable gas exchange system (LI-6400XT). Values are means ± SE (*n*=three independent experiments, each with six plants per genotype). ^*^p<0.05; ^**^p<0.01; ns, no significant difference; Student’s *t*-test (**E, F**), and different letters indicate statistically significant differences (*p*<0.05) by one-way ANOVA and Tukey’s test analyses (**G, H**). Quantification of leaf temperature by the software ThermaCAM Researcher Professional 2.10 (**B**, **D**).

To explore the function of *GGLs* in stomatal biology, we ordered T-DNA insertion mutants of seven guard cell preferentially expressed *GGLs* (Figures 2B, 3) from ABRC stock, which were speculated to have specific roles in stomata. Genotyping and RT-PCR analyses showed that *ggl12*, *ggl14*, *ggl22*, and *ggl27* were knockout mutants, and *ggl7* and *ggl26* were knockdown mutants (Supplementary Figure S5B). However, the expression level of *GGL23* was not changed in the *ggl23* mutant (Supplementary Figure S5B). Therefore, the *ggl23* mutant was not used for further analyses in this study. We firstly used thermal imaging to detect the leaf temperature of these six single mutants, which reflects the transpiration efficiency through the stomatal pores and epidermis. Thermal imaging analyses revealed that *ggl14* mutant (*osp1-1*) exhibited higher leaf temperature, consistent with our previous study (Tang et al., 2020), and *ggl22* mutant exhibited lower leaf temperature than Col-0, whereas the remaining four mutants showed comparable leaf temperatures as Col-0 (Figures 6A,B). To determine whether there is functional redundancy between *GGL14* with the other guard cell preferentially expressed *GGLs*, *ggl14* was crossed with *ggl7* and *ggl26* to generate double and triple mutants since these three genes are relatively higher and specifically expressed in guard cells than other *GGLs*, and are coexpressed with known components that function in stomata by coexpression analyses (Obayashi et al., 2009). *ggl7ggl14* and *ggl14ggl26* showed similar leaf temperatures as *ggl14*, and *ggl7ggl26* behaved WT-like leaf temperature (Figures 6C,D). However, the *ggl7ggl14ggl26* triple mutant showed significantly higher leaf temperature than *ggl14* and double mutants (Figures 6C,D), suggesting that GGL7, GGL14, and GGL26 have functional redundancy in transpiration, and GGL14 is a major contributor in this process. We next measured the transpiration rate and WUE of these single, double, and triple mutant plants. *ggl14* mutant exhibited a reduced transpiration rate and increased WUE than Col-0, while *ggl22* had an increased transpiration rate than Col-0 (Figures 6E,F), in accordance with their leaf temperatures (Figures 6A,B). Consistently, the transpiration rate of *ggl7ggl14ggl26* triple mutant was further reduced, and the increase of WUE in *ggl7ggl14ggl26* was aggravated compared to *ggl14* (Figures 6G,H), further supporting the functional redundancy among GGL7, GGL14, and GGL26.

**Figure 7:**
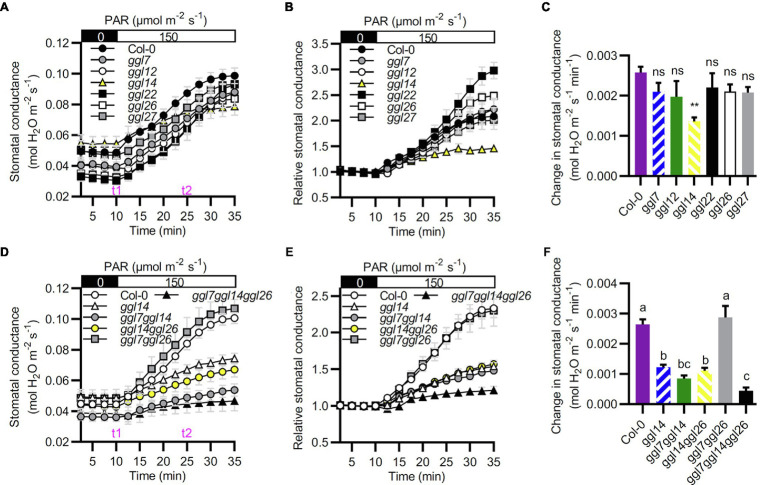
*GGL7*, *GGL14*, and *GGL26* show functional redundancy in stomatal dynamics during the dark-to-light transition. (**A–C**) Time-resolved stomatal conductance responses to dark to light transitions in Col-0 and *ggl* single mutant plants. (**B**) Relative stomatal conductance. Normalized stomatal conductance of (**A**). (**C**) The initial rates of stomatal conductance changes in the period of t1 to t2 in (**A**), presented as mol H_2_O m^−2^ s^−1^ min^−1^. Values are means ± SE (*n*=3 independent experiments, each with at least four leaves per genotype). ^**^p<0.01; ns, no significant difference; Student’s *t*-test. (**D–F**) Time-resolved stomatal conductance responses to dark to light transitions in Col-0, *ggl14*, *ggl7ggl14*, *ggl14ggl26*, *ggl7ggl26*, and *ggl7ggl14ggl26* mutant plants. (**E**) Relative stomatal conductance. Normalized stomatal conductance of (**D**). (**F**) The initial rates of stomatal conductance changes in the time of t1 to t2 in (**D**), presented as mol H_2_O m^−2^ s^−1^ min^−1^. Values are means ± SE (*n*=3 independent experiments, each with at least four leaves per genotype). Different letters indicate statistically significant differences (*p*<0.05) by one-way ANOVA and Tukey’s test analyses. PAR: photosynthetically active radiation.

We then detected their stomatal dynamics to dark-to-light transitions to determine whether these six *GGLs* are involved in stomatal dynamics when responses to environmental changes. *ggl14* exhibited impaired light-induced stomatal opening (Figures 7A–C), in agreement with our previous study (Tang et al., 2020), and the other single mutants retained intact stomatal response (Figures 7A–C). However, *ggl22* and *ggl26* single mutants exhibited relatively larger stomatal conductance when the stomatal aperture reached maximum value (Figure 7B), indicating that mutation of *GGL22* or *GGL26* increased stomatal movement capacity but not the stomatal sensitivity (Hu et al., 2015). To explore whether other GGLs have functional redundancy with GGL14 in stomatal dynamics to dark-to-light transitions, we also investigated the stomatal response of double and triple mutant plants to dark-to-light transitions. Similar to transpiration rate and WUE (Figure 6), *ggl7ggl14* and *ggl14ggl26* had similar stomatal dynamics as *ggl14*, which was greatly impaired compared to Col-0 (Figures 7D–F). However, the impairment in the light-induced stomatal opening was aggravated in *ggl7ggl14ggl26* triple mutant compared to *ggl14* (Figures 7D–F). These results suggest that GGL7, GGL14, and GGL26 are redundant in stomatal responses, at least to dark-to-light transitions and water maintenance.

### Mutation of *GGLs* Affects Stomatal Density and Stomatal Morphology

**Figure 8:**
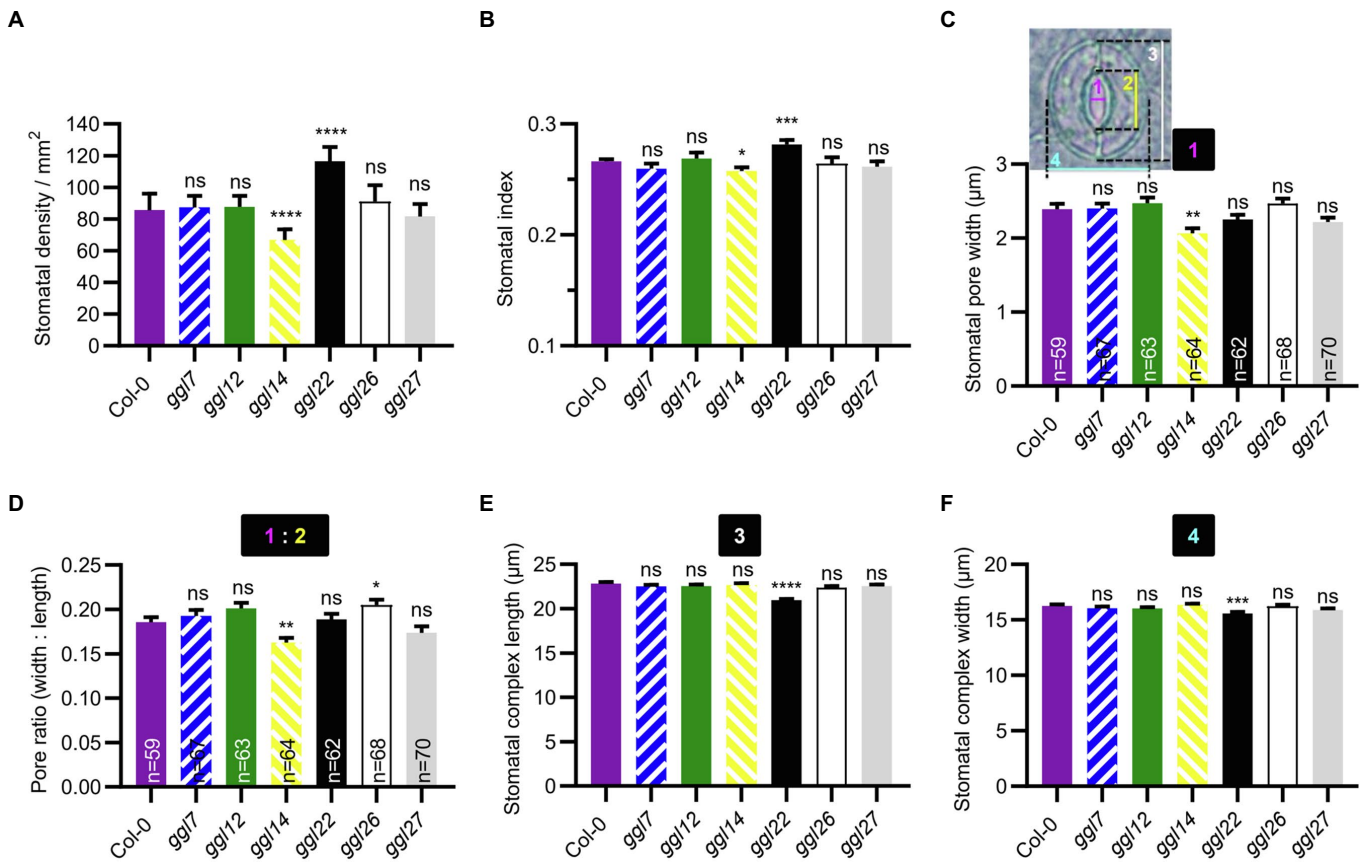
Stomatal density and stomatal morphology of *ggl* single mutants. (**A, B**) Stomatal density (**A**) and stomatal index (**B**) in the abaxial leaves of Col-0 and *ggl* single mutants. Values are means ± SE (*n*=3 independent experiments, each with at least 8 leaves per genotype). ^*^p<0.05; ^***^p<0.001; ^****^p<0.0001; ns, no significant difference; Student’s *t*-test. (**C–F**) Stomatal pore width (**C**), pore ratio (width: length) (**D**), stomatal complex length (**E**), and width (**F**) of Col-0 and *ggl* single mutants. Number 1 represents stomatal pore width, 2 represents stomatal pore length, 3 represents stomatal complex length, and 4 represents stomatal complex width. Pore ratio (1:2) is the ratio of pore width to pore length. Data are means ± SE, *n*=60 stomata from at least six leaves per genotype (**E**) and (**F**). ^*^p<0.05; ^**^p<0.01; ^***^p<0.001; ^****^p<0.0001; ns, no significant difference; Student’s *t*-test. The measurement of stomatal pore width and length, and stomatal complex length and width was indicated in (**C**).

We were also interested in whether these *GGLs* played roles in stomatal density and stomatal morphology. The stomatal density and index of *ggl22* were significantly increased than those in Col-0, and *ggl14* showed reduced stomatal density and index than Col-0 (Figures 8A,B). These results suggest that GGL22 is a negative regulator and GGL14 is a positive one to mediate stomatal density. Moreover, the stomatal density and index of *ggl7ggl14ggl26* triple mutant were not different from those in *ggl14* (Supplementary Figures S6A,B), suggesting that GGL7 and GGL26 are not involved in stomatal density. It has been reported that some guard cell-expressed genes affect stomatal patterning and shape (Lee et al., 2013; Negi et al., 2013; Castorina et al., 2016; Rui et al., 2017). We found that the one-spacing rule in these single, double, and triple mutants was not disrupted (data not shown), suggesting that these GGLs are not involved in this stomatal developmental process.

Furthermore, stomatal pore width, length, and stomatal complex size were measured in these mutants at normal growth conditions. The stomatal pore width and the width to length ratio (pore ratio) of *ggl14* were significantly smaller than Col-0 (Figures 8C,D), partially explaining the higher leaf temperature of *ggl14* mutant (Figures 6A,B). Although the stomatal pore width of *ggl26* was not obviously different from that in Col-0, its pore ratio was greater than Col-0 (Figures 8C,D). Measurement of stomatal complex length and width revealed that *ggl22* had a smaller stomatal complex size than Col-0, while the other five *ggl* single mutants showed a comparable stomatal complex size as Col-0 (Figures 8E,F). These results suggest that GGL26 and GGL22 influence stomatal pore dimension and stomatal complex size, respectively. *ggl7ggl14ggl26* phenocopied *ggl14* with respect to stomatal pore width and pore ratio (Supplementary Figures S6C,D), indicating GGL7 and GGL26 do not show functional redundancy with GGL14 in this developmental process. We interestingly found that *ggl7ggl14ggl26* had a larger stomatal complex length, whereas their corresponding single mutants showed a similar length as Col-0 (Figure 8E and Supplementary Figure S6E). These results suggest that GGL7, GGL14, and GGL26 are required and show redundancy in keeping stomatal complex at suitable size during development.

### Mutation of *GGLs* Affects Plant Drought Performance

Environmental changes affect stomatal status and stomatal development. The public database (AtGenExpress Visualization Tool) showed that some *GGLs* were hormone or abiotic stress-inducible (Kilian et al., 2007). To further determine their expression profiles, we determined the expression patterns of these 19 *GGLs* during hormone or stress treatments by RT-PCR (Supplementary Figures S7, S8). The results revealed that *GGL13*, *GGL21*, and *GGL23* were upregulated by ABA treatment (Supplementary Figure S7). *GGL13* showed dynamic responses to IAA treatment, and IAA treatment inhibited *GGL21* expression (Supplementary Figure S7). *GGL27* was repressed by BL treatment and accumulated by GA treatment (Supplementary Figure S7). GA treatment inhibited the expression of *GGL8*, *GGL12*, and *GGL26* (Supplementary Figure S7). *GGL5* and *GGL22* were prominently downregulated, and *GGL7* was activated during the process of salt treatment (Supplementary Figure S8A), whereas *GGL6*, *GGL8*, *GGL12*, *GGL13*, *GGL14*, *GGL16*, *GGL17*, and *GGL26* showed dynamic changes during salt treatment (Supplementary Figure S8A). Under dehydration stresses, the expression levels of *GGL5*, *GGL6*, *GGL16*, *GGL19*, *GGL22*, *GGL23*, and *GGL29* were downregulated (Supplementary Figure S8B), whereas another four *GGLs* (*GGL8*, *GGL13*, *GGL14*, and *GGL17*) were significantly upregulated at different time points under dehydration stresses (Supplementary Figure S8B). Furthermore, the expression of six *GGLs* (*GGL7*, *GGL9*, *GGL12*, *GGL21*, *GGL26*, and *GGL27*) increased first and then decreased during dehydration treatment (Supplementary Figure S8B). To further confirm these results, we determined expression patterns of several hormone or stress-inducible *GGLs* (Supplementary Figures S7, S8) by real-time quantitative PCR. Our qPCR analyses showed that the expression patterns of these selected *GGLs* in response to hormones, salt, or dehydration stresses were generally consistent with RT-PCR results (Supplementary Figure S9). These results indicate that *GGLs* are more inducible to drought stresses and that hormone or stress-inducible *GGLs* might be involved in plant development and adaptation to stresses.

**Figure 9:**
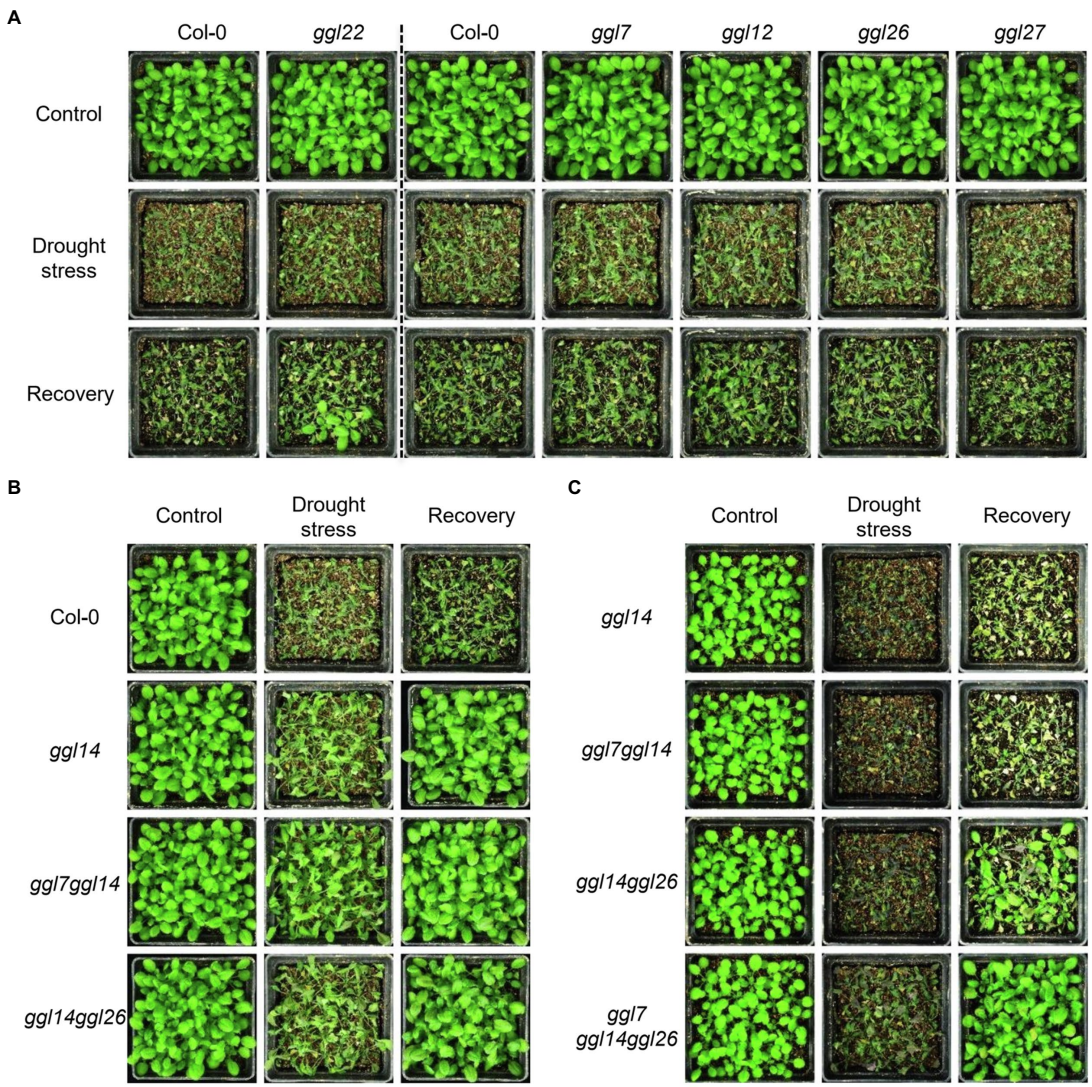
*GGL7*, *GGL14*, and *GGL26* show functional redundancy in plant drought performance. (**A**) Drought performance of *ggl7*, *ggl12*, *ggl22*, *ggl26*, and *ggl27* single mutants under moderate drought stresses. (**B**) Drought performance of *ggl14*, *ggl7ggl14*, and *ggl14ggl26* mutant plants under moderate drought stresses. (**C**) Drought performance of *ggl14*, *ggl7ggl14*, *ggl14ggl26*, and *ggl7ggl14ggl26* under severe drought stresses. Twenty-five plants per genotype were grown in the pots containing the same weight of soil and water content. For moderate drought stresses, plants were rewatered when *ggl14* and WT plants showed significantly different wilting phenotypes. For severe drought stresses, plants were rewatered when significantly different wilting phenotypes were observed in *ggl14* and *ggl7ggl14ggl26* plants. The experiment was repeated two times with similar results.

To test the effects of *GGLs* on drought performance, we subjected six *ggl* single mutants and the double and triple mutant plants to drought stresses. Under moderate drought stresses, *ggl14* showed greatly enhanced drought tolerance (Figure 9B), consistent with WUE (Figure 6F) and our previous report (Tang et al., 2020). *ggl22* showed slightly increased drought recovery capacity (Figure 9A). The rest four *ggl* single mutants performed the same drought performance as Col-0 (Figure 9A). *ggl7ggl14* and *ggl14ggl26* double mutants behaved similar drought performance as *ggl14* (Figure 9B). Under severe drought stresses, *ggl14ggl26* showed enhanced drought tolerance than *ggl14* mutant plants, and the drought tolerance in *ggl7ggl14ggl26* triple mutant was much stronger than *ggl14ggl26* (Figure 9C), suggesting GGL7, GGL14, and GGL26 have redundancy in water maintenance.

## Discussion

Plants encounter many environmental changes and have to deal with these badly living conditions for survival by triggering different cellular responses. Stomata respond quickly to these environmental changes. GDSL lipases exist as a big family in most plant species, and more than 100 members have been identified in different plant species (Ling, 2008; Chepyshko et al., 2012; Dong et al., 2016; Lai et al., 2017; Su et al., 2020). However, only a few members have been studied for their biological and biochemical functions, especially in stomatal biology, though GDSL lipases/esterases that identified play essential roles in many aspects, such as regulation of plant growth, development, and stress adaptations (Naranjo et al., 2006; Hong et al., 2008; Takahashi et al., 2010; Girard et al., 2012; Gao et al., 2017; An et al., 2019).

It has been suggested that gene function is highly correlated with its expression pattern (Wu et al., 2016). For instance, two flower-expressed *GDSLs* (*EXL4* and *CDEF1*) promoted pollen hydration on the stigma and facilitated pollen tube penetration into the stigma, respectively (Updegraff et al., 2009; Takahashi et al., 2010). In the present study, we firstly isolated 29 putative guard cell highly expressed *GDSLs* (here named *GGLs*) through the published microarray data (Leonhardt et al., 2004; Yang et al., 2008) and confirmed the expression patterns of 19 *GGLs* in L clade by *GGL*pro::*GUS* analyses (Figure 1). *GGL6* (*GELP16*/*GLIP9*/*AtGDSL1*) and *GGL22* (*GELP80*/*SFAR5*) were highly expressed in the seed germination stage (Figure 2B and Table 1), consistent with previous studies (Chen et al., 2012; Ding et al., 2019), suggesting our system works well. Nineteen *GGLs* showed diverse expression patterns during the whole plant growth stages. Fifteen of them were confirmed to express in leaf guard cells, and seven (*GGL7*, *GGL12*, *GGL14*, *GGL17*, *GGL23*, *GGL26*, and *GGL27*) were preferentially expressed in leaf guard cells (Figures 2B, 3). These results indicate the potential roles of these GGLs in stomatal biology and the possibility of functional redundancy among them.

**Table 1:** Gene name and AGI gene code comparison.

Gene name	AGI Gene Code	Gene name	Reference
*GGL5*	AT1G29660	*AtGELP15 (AED4)*	Breitenbach et al. (2014) and Lai et al. (2017)
*GGL6*	AT1G29670	*AtGELP16 (GLIP9/AtGDSL1)*	Lai et al. (2017) and Ding et al. (2019)
*GGL7*	AT1G33811	*AtGELP18*	Lai et al. (2017)
*GGL8*	AT1G53920	*AtGELP19 (GLIP5)*	Lai et al. (2017)
*GGL9*	AT1G54000	*AtGELP22 (GLL22)*	Lai et al. (2017)
*GGL12*	AT1G75880	*AtGELP39 (EXL1)*	Lai et al. (2017)
*GGL13*	AT1G75900	*AtGELP41 (EXL3)*	Lai et al. (2017)
*GGL14*	AT2G04570	*AtGELP47 (OSP1)*	Lai et al. (2017) and Tang et al. (2020)
*GGL16*	AT3G05180	*AtGELP61 (AED5)*	Breitenbach et al. (2014) and Lai et al. (2017)
*GGL17*	AT3G11210	GELP pseudoenzyme	Dong et al. (2016)
*GGL19*	AT3G16370	*AtGELP67*	Lai et al. (2017)
*GGL20*	AT3G26430	*AtGELP68*	Lai et al. (2017)
*GGL21*	AT3G48460	*AtGELP72 (SFAR4)*	Lai et al. (2017)
*GGL22*	AT4G18970	*AtGELP80 (SFAR5)*	Lai et al. (2017)
*GGL23*	AT4G26790	*AtGELP81*	Lai et al. (2017)
*GGL25*	AT5G03610	*AtGELP86*	Lai et al. (2017)
*GGL26*	AT5G18430	*AtGELP93*	Lai et al. (2017)
*GGL27*	AT5G45920	GELP pseudoenzyme	Dong et al. (2016)
*GGL29*	AT5G62930	GELP pseudoenzyme	Dong et al. (2016)

The roles of GGLs in stomata were further confirmed by phenotypic identification of T-DNA insertion mutants of six guard cell preferentially expressed *GGLs*. Our previous study has shown that *OSP1*/*GGL14* plays an essential role in stomata (Tang et al., 2020). Here, we identified the functional redundancy of GGL7 and GGL26 with GGL14 in modulating transpiration, WUE, and stomatal dynamics (Figures 6, 7, 9), but not in stomatal density and stomatal pore dimension (Supplementary Figures S6A–D). Our study shows that GGL14 and GGL26 play essential roles in regulating the pore dimension. Mutation of *GGL14* or *GGL26* influenced the size of stomatal pores with opposite effects (Figures 8C,D). However, *ggl7ggl14ggl26* and *ggl14* had similar pore width and pore ratio (Supplementary Figures S6C,D), which may be due to the major role of GGL14 in this aspect. In addition, GGL7, GGL14, and GGL26 also have a role in controlling stomatal complex length with redundancy (Figure 8E and Supplementary Figure S6E). These results suggest that GGLs have functional similarity but also specificity in stomatal development and stomatal behavior.

Our study also suggests that GGL22 is a component involved in stomatal biology. *GGL22* mutation increased stomatal density and stomatal index (Figures 8A,B), partially explaining the increased transpiration rate and reduced leaf temperature than Col-0 at normal growth conditions (Figures 6A,B,E). However, *ggl22* exhibited increased stomatal movement capacity and reduced stomatal complex size (Figures 7B, 8E,F), which may account for the slightly increased drought tolerance compared to Col-0 (Figure 9A). Given that mutation of *GGLs* affects stomatal density, stomatal pore dimension, and stomatal complex size, whether these *GGLs* control stomatal development and the underlying mechanism need to be further investigated in the future. Moreover, in these guard cell preferentially expressed *GGLs*, others may also be involved in stomatal biology if more double or triple mutants are generated and investigated according to our expression pattern data. Our present investigations further support the correlation between the expression pattern and biological function, and also suggest that investigation of expression patterns of genes gives valuable and vital information for determining their functions.

Five *GGLs* (*GGL5*, *GGL14*, *GGL17*, *GGL19*, and *GGL23*) showed expressions in trichomes (Figure 3 and Supplementary Figure S2), and seven *GGLs* (*GGL6*, *GGL8*, *GGL9*, *GGL13*, *GGL16*, *GGL22*, and *GGL29*) were also expressed in the vascular tissues of true leaves (Figure 3). These results imply that these *GGLs* may play vital roles in trichome or vascular tissue development. Moreover, most of these 19 *GGLs* were expressed in the floral organ of 34-DAG plants (Figure 4), indicating these *GGLs* may also be involved in regulating floral organ development or fertility, possibly with redundancy. The deficiency in early siliques fertility and trichome development in the *osp1* mutants (Tang et al., 2020) and an increasing number of reports showing that GDSLs play important roles in regulating plants fertility (Huo et al., 2020; Zhao et al., 2020; Zhu et al., 2020) support our conclusions. During the whole plant growth process, the GUS signal of *GGL20*pro::*GUS* expressing plants was not detected (Figures 2B, 3, 4). It may be due that *GGL20* contains a long 5′ UTR (Supplementary Figure S4), which regulates its basal expression (Broad et al., 2019; Nitschke et al., 2020), or the upstream sequence of ATG we obtained may not include the intact promoter of *GGL20*. Our expression profile analyses revealed that some *GGLs* were inducible by hormones (Supplementary Figure S7), and most of them were influenced by dehydration (Supplementary Figure S8B), suggesting that they may have essential roles in plant development, adaptation to environmental changes, and hormone treatment. During dehydration, *GGL22* was downregulated, and *GGL14* was activated, whereas *GGL7* and *GGL26* showed dynamic responses (Supplementary Figure S8B). The functions of these four GGLs in stomatal biology and plant drought performance were validated in this study (Figures 7–9) and our previous report (Tang et al., 2020). These results further support the correlation of expression patterns and biological functions. The roles of other GGLs in abiotic stresses and hormone pathways need to be further investigated.

Proteins are distributed in different cell compartments to fulfill their diverse biological functions. In the present study, we found that GGL5, GGL8, GGL13, GGL14, GGL16, GGL17, GGL20, GGL27, and GGL29 were localized in ER (Figure 5A). A previous study has shown that GGL5 is not located in ER (Barbaglia et al., 2016). The difference in its location between the two labs may be due to the fact that only a tiny amount of GGL5 in ER can only be monitored by a high-resolution confocal microscope, or GGL5-YFP controlled by a *35S* promoter leads to an artifact in *N. benthamiana* leaf epidermis. Together with that some GGLs were located in lipid droplets (Figure 5 and Supplementary Figure S3), these results imply that GGLs may function in stomata through regulating lipid biosynthesis and homeostasis. The previous studies revealed that the eukaryotic lipid metabolic pathway and the breakdown of stored triacylglycerols (TAGs) are essential for stomatal response to light intensity changes in *Arabidopsis* guard cells (McLachlan et al., 2016; Negi et al., 2018). Recently, more and more reports have shown that biochemical enzymes have other functions. For example, rice aldehyde dehydrogenase ALDH2B1 and rice glyceraldehyde-3-phosphatedehydrogenase (GAPDH) also act as transcriptional regulators to regulate gene expression (Zhang et al., 2017b; Ke et al., 2020). We found that four GGLs were localized in nucleus (Figures 5A,C and Supplementary Figure S3), suggesting that GGLs may have some special roles in nucleus. The diverse expression patterns and subcellular localization suggest that these GGLs may have diverse functions in plant development and environmental adaptations.

The previous phylogenetic analysis classified the *Arabidopsis* GDSLs into four clades (Lai et al., 2017). Our phylogenetic analyses of these 19 GGLs suggest that members of GGLs with high homology show similar tissue or subcellular expression patterns. GGL6 and GGL22, and GGL14 and GGL23 had mostly closed homologies, respectively, and showed similar expression patterns in most plant tissues (Figures 2B, 3, 4 and Supplementary Figure S2). GGL14 and GGL22 are involved in stomatal biology (Figures 7, 8). GGL6 and GGL23 may also have some roles in stomata, which need to be further determined. GGL17, GGL27, and GGL29 showed high homology and displayed the same subcellular localization (Figure 5A and Supplementary Figures S3A,C), indicating the similarity of functions among them.

In conclusion, we systematically investigated the expression patterns of 19 *GGLs* in *Arabidopsis*. Our results showed that most of these *GGLs* exhibited consistent expression patterns under normal growth conditions. At the cellular level, seven *GGLs* were preferentially, and eight were highly expressed in leaf guard cells. Expression pattern analyses under dehydration and phenotypic identification of mutants revealed a high correlation between expression pattern and biological function, and functional redundancy among the genes with similar expression patterns. Our findings also showed that protein sequence similarity had some degree of correlation with tissue or subcellular expression patterns. These findings provide valuable resources for future functional analyses of these GGLs in stomatal biology and developmental processes.

## Data Availability Statement

The original contributions presented in the study are included in the article/Supplementary Material; further inquiries can be directed to the corresponding author.

## Author Contributions

HH and CX conceived and designed the research, analyzed the data, and wrote the paper. CX, HG, JT, and JL performed the experiments. XY discussed the data. All authors contributed to the article and approved the submitted version.

## Funding

This work was supported by grants from the National Science Foundation of China (31970730 and 31771552) and the National Key Research and Development Program (2016YFD0100604).

## Conflict of Interest

The authors declare that the research was conducted in the absence of any commercial or financial relationships that could be construed as a potential conflict of interest.

## Publisher’s Note

All claims expressed in this article are solely those of the authors and do not necessarily represent those of their affiliated organizations, or those of the publisher, the editors and the reviewers. Any product that may be evaluated in this article, or claim that may be made by its manufacturer, is not guaranteed or endorsed by the publisher.

## Footnotes

^1^ https://www.arabidopsis.org/jsp/ChromosomeMap/tool.jsp

^2^ http://www.cbs.dtu.dk/services/SignalP/

^3^ www.arabidopsis.org

## References

[ref1] Akoh C. C. Lee G. C. Liaw Y. C. Huang T. H. Shaw J. F. (2004). GDSL family of serine esterases/lipases. Prog. Lipid Res. 43, 534–552. doi: 10.1016/j.plipres.2004.09.002, PMID: 15522763

[ref2] An X. Dong Z. Tian Y. Xie K. Wu S. Zhu T.. (2019). ZmMs30 encoding a novel GDSL lipase is essential for male fertility and valuable for hybrid breeding in maize. Mol. Plant 12, 343–359. doi: 10.1016/j.molp.2019.01.011, PMID: 30684599

[ref3] Barbaglia A. M. Tamot B. Greve V. Hoffmann-Benning S. (2016). Phloem proteomics reveals new lipid-binding proteins with a putative role in lipid-mediated signaling. Front. Plant Sci. 7:563. doi: 10.3389/fpls.2016.00563, PMID: 27200036 PMC4849433

[ref001] Breitenbach H. H. Wenig M. Wittek F. Jordá L. Maldonado-Alconada A. M. Sarioglu H.. (2014). Contrasting Roles of the Apoplastic Aspartyl Protease APOPLASTIC, ENHANCED DISEASE SUSCEPTIBILITY1-DEPENDENT1 and LEGUME LECTIN-LIKE PROTEIN1 in Arabidopsis Systemic Acquired Resistance. Plant Physiol. 165, 791–809. doi: 10.1104/pp.114.239665 24755512 PMC4044859

[ref4] Broad R. C. Bonneau J. P. Beasley J. T. Roden S. Philips J. G. Baumann U.. (2019). Genome-wide identification and characterization of the GDP-L-galactose phosphorylase gene family in bread wheat. BMC Plant Biol. 19:515. doi: 10.1186/s12870-019-2123-1, PMID: 31771507 PMC6878703

[ref5] Castorina G. Fox S. Tonelli C. Galbiati M. Conti L. (2016). A novel role for STOMATAL CARPENTER 1 in stomata patterning. BMC Plant Biol. 16:172. doi: 10.1186/s12870-016-0851-z, PMID: 27484174 PMC4970199

[ref6] Charrier B. Leroux C. Kondorosi A. Ratet P. (1996). The expression pattern of alfalfa flavanone 3-hydroxylase promoter-gus fusion in *Nicotiana benthamiana* correlates with the presence of flavonoids detected in situ. Plant Mol. Biol. 30, 1153–1168. doi: 10.1007/BF00019549, PMID: 8704126

[ref7] Chen C. Chen H. Zhang Y. Thomas H. R. Frank M. H. He Y.. (2020). TBtools: An integrative toolkit developed for interactive analyses of big biological data. Mol. Plant 13, 1194–1202. doi: 10.1016/j.molp.2020.06.009, PMID: 32585190

[ref8] Chen M. Du X. Zhu Y. Wang Z. Hua S. Li Z.. (2012). Seed fatty acid reducer acts downstream of gibberellin signalling pathway to lower seed fatty acid storage in Arabidopsis. Plant Cell Environ. 35, 2155–2169. doi: 10.1111/j.1365-3040.2012.02546.x, PMID: 22632271

[ref9] Chepyshko H. Lai C. P. Huang L. M. Liu J. H. Shaw J. F. (2012). Multifunctionality and diversity of GDSL esterase/lipase gene family in rice (Oryza sativa L. japonica) genome: new insights from bioinformatics analysis. BMC Genomics 13:309. doi: 10.1186/1471-2164-13-309, PMID: 22793791 PMC3412167

[ref10] Clauss K. Baumert A. Nimtz M. Milkowski C. Strack D. (2008). Role of a GDSL lipase-like protein as sinapine esterase in Brassicaceae. Plant J. 53, 802–813. doi: 10.1111/j.1365-313X.2007.03374.x, PMID: 18036206

[ref11] Clauss K. von Roepenack-Lahaye E. Bottcher C. Roth M. R. Welti R. Erban A.. (2011). Overexpression of sinapine esterase BnSCE3 in oilseed rape seeds triggers global changes in seed metabolism. Plant Physiol. 155, 1127–1145. doi: 10.1104/pp.110.169821, PMID: 21248075 PMC3046574

[ref12] Curtis M. D. Grossniklaus U. (2003). A gateway cloning vector set for high-throughput functional analysis of genes in planta. Plant Physiol. 133, 462–469. doi: 10.1104/pp.103.027979, PMID: 14555774 PMC523872

[ref13] Deeks M. J. Calcutt J. R. Ingle E. K. Hawkins T. J. Chapman S. Richardson A. C.. (2012). A superfamily of actin-binding proteins at the actin-membrane nexus of higher plants. Curr. Biol. 22, 1595–1600. doi: 10.1016/j.cub.2012.06.041, PMID: 22840520

[ref14] Ding L. N. Guo X. J. Li M. Fu Z. L. Yan S. Z. Zhu K. M.. (2019). Improving seed germination and oil contents by regulating the GDSL transcriptional level in Brassica napus. Plant Cell Rep. 38, 243–253. doi: 10.1007/s00299-018-2365-7, PMID: 30535511

[ref15] Dong X. Yi H. Han C. T. Nou I. S. Hur Y. (2016). GDSL esterase/lipase genes in Brassica rapa L.: genome-wide identification and expression analysis. Mol. Gen. Genomics. 291, 531–542. doi: 10.1007/s00438-015-1123-6, PMID: 26423069

[ref16] Dunn J. Hunt L. Afsharinafar M. Meselmani M. A. Mitchell A. Howells R.. (2019). Reduced stomatal density in bread wheat leads to increased water-use efficiency. J. Exp. Bot. 70, 4737–4748. doi: 10.1093/jxb/erz248, PMID: 31172183 PMC6760291

[ref17] Feng Z. Zhang B. Ding W. Liu X. Yang D. L. Wei P.. (2013). Efficient genome editing in plants using a CRISPR/Cas system. Cell Res. 23, 1229–1232. doi: 10.1038/cr.2013.114, PMID: 23958582 PMC3790235

[ref18] Gao M. Yin X. Yang W. Lam S. M. Tong X. Liu J.. (2017). GDSL lipases modulate immunity through lipid homeostasis in rice. PLoS Pathog. 13:e1006724. doi: 10.1371/journal.ppat.1006724, PMID: 29131851 PMC5703576

[ref19] Girard A. L. Mounet F. Lemaire-Chamley M. Gaillard C. Elmorjani K. Vivancos J.. (2012). Tomato GDSL1 is required for cutin deposition in the fruit cuticle. Plant Cell 24, 3119–3134. doi: 10.1105/tpc.112.101055, PMID: 22805434 PMC3426136

[ref20] Hauser F. Chen W. Deinlein U. Chang K. Ossowski S. Fitz J.. (2013). A genomic-scale artificial microRNA library as a tool to investigate the functionally redundant gene space in Arabidopsis. Plant Cell 25, 2848–2863. doi: 10.1105/tpc.113.112805, PMID: 23956262 PMC3784584

[ref21] Hong J. K. Choi H. W. Hwang I. S. Kim D. S. Kim N. H. Choi D. S.. (2008). Function of a novel GDSL-type pepper lipase gene, CaGLIP1, in disease susceptibility and abiotic stress tolerance. Planta 227, 539–558. doi: 10.1007/s00425-007-0637-5, PMID: 17929052

[ref22] Hu H. Boisson-Dernier A. Israelsson-Nordstrom M. Bohmer M. Xue S. Ries A.. (2010). Carbonic anhydrases are upstream regulators of CO_2_-controlled stomatal movements in guard cells. Nat. Cell Biol. 12, 87–93. doi: 10.1038/ncb2009, PMID: 20010812 PMC2906259

[ref23] Hu H. Rappel W. J. Occhipinti R. Ries A. Bohmer M. You L.. (2015). Distinct cellular locations of carbonic anhydrases mediate carbon dioxide control of stomatal movements. Plant Physiol. 169, 1168–1178. doi: 10.1104/pp.15.00646, PMID: 26243620 PMC4587455

[ref24] Huang S. Ding M. Roelfsema M. R. G. Dreyer I. Scherzer S. Al-Rasheid K. A. S.. (2021). Optogenetic control of the guard cell membrane potential and stomatal movement by the light-gated anion channel GtACR1. Sci. Adv. 7:eabg4619. doi: 10.1126/sciadv.abg4619, PMID: 34244145 PMC8270491

[ref25] Hughes J. Hepworth C. Dutton C. Dunn J. A. Hunt L. Stephens J.. (2017). Reducing stomatal density in barley improves drought tolerance without impacting on yield. Plant Physiol. 174, 776–787. doi: 10.1104/pp.16.01844, PMID: 28461401 PMC5462017

[ref26] Huo Y. Pei Y. Tian Y. Zhang Z. Li K. Liu J.. (2020). IRREGULAR POLLEN EXINE2 encodes a GDSL lipase essential for male fertility in maize. Plant Physiol. 184, 1438–1454. doi: 10.1104/pp.20.00105, PMID: 32913046 PMC7608179

[ref27] Ke Y. Yuan M. Liu H. Hui S. Qin X. Chen J.. (2020). The versatile functions of OsALDH2B1 provide a genic basis for growth-defense trade-offs in rice. Proc. Natl. Acad. Sci. U. S. A. 117, 3867–3873. doi: 10.1073/pnas.1918994117, PMID: 32024752 PMC7035479

[ref28] Kilian J. Whitehead D. Horak J. Wanke D. Weinl S. Batistic O.. (2007). The AtGenExpress global stress expression data set: protocols, evaluation and model data analysis of UV-B light, drought and cold stress responses. Plant J. 50, 347–363. doi: 10.1111/j.1365-313X.2007.03052.x, PMID: 17376166

[ref29] Korkuæ P. Schippers J. H. Walther D. (2014). Characterization and identification of cis-regulatory elements in Arabidopsis based on single-nucleotide polymorphism information. Plant Physiol. 164, 181–200. doi: 10.1104/pp.113.229716, PMID: 24204023 PMC3875800

[ref30] Kwon S. J. Jin H. C. Lee S. Nam M. H. Chung J. H. Kwon S. I.. (2009). GDSL lipase-like 1 regulates systemic resistance associated with ethylene signaling in Arabidopsis. Plant J. 58, 235–245. doi: 10.1111/j.1365-313X.2008.03772.x, PMID: 19077166

[ref31] Lai C. P. Huang L. M. Chen L. O. Chan M. T. Shaw J. F. (2017). Genome-wide analysis of GDSL-type esterases/lipases in Arabidopsis. Plant Mol. Biol. 95, 181–197. doi: 10.1007/s11103-017-0648-y, PMID: 28840447

[ref32] Lee E. Liu X. Eglit Y. Sack F. (2013). FOUR LIPS and MYB88 conditionally restrict the G1/S transition during stomatal formation. J. Exp. Bot. 64, 5207–5219. doi: 10.1093/jxb/ert313, PMID: 24123248 PMC3830495

[ref33] Leonhardt N. Kwak J. M. Robert N. Waner D. Leonhardt G. Schroeder J. I. (2004). Microarray expression analyses of Arabidopsis guard cells and isolation of a recessive abscisic acid hypersensitive protein phosphatase 2C mutant. Plant Cell 16, 596–615. doi: 10.1105/tpc.019000, PMID: 14973164 PMC385275

[ref34] Ling H. (2008). Sequence analysis of GDSL lipase gene family in Arabidopsis thaliana. Pak. J. Biol. Sci. 11, 763–767. doi: 10.3923/pjbs.2008.763.767, PMID: 18819574

[ref35] Ling H. Zhao J. Zuo K. Qiu C. Yao H. Qin J.. (2006). Isolation and expression analysis of a GDSL-like lipase gene from Brassica napus L. J. Biochem. Mol. Biol. 39, 297–303. doi: 10.5483/bmbrep.2006.39.3.297, PMID: 16756759

[ref36] Mayfield J. A. Fiebig A. Johnstone S. E. Preuss D. (2001). Gene families from the Arabidopsis thaliana pollen coat proteome. Science 292, 2482–2485. doi: 10.1126/science.1060972, PMID: 11431566

[ref37] McLachlan D. H. Lan J. Geilfus C. M. Dodd A. N. Larson T. Baker A.. (2016). The breakdown of stored triacylglycerols is required during light-induced stomatal opening. Curr. Biol. 26, 707–712. doi: 10.1016/j.cub.2016.01.019, PMID: 26898465 PMC4791430

[ref38] Miao J. Guo D. Zhang J. Huang Q. Qin G. Zhang X.. (2013). Targeted mutagenesis in rice using CRISPR-Cas system. Cell Res. 23, 1233–1236. doi: 10.1038/cr.2013.123, PMID: 23999856 PMC3790239

[ref39] Nakagawa T. Suzuki T. Murata S. Nakamura S. Hino T. Maeo K.. (2007). Improved gateway binary vectors: high-performance vectors for creation of fusion constructs in transgenic analysis of plants. Biosci. Biotechnol. Biochem. 71, 2095–2100. doi: 10.1271/bbb.70216 17690442

[ref40] Naranjo M. A. Forment J. Roldan M. Serrano R. Vicente O. (2006). Overexpression of Arabidopsis thaliana LTL1, a salt-induced gene encoding a GDSL-motif lipase, increases salt tolerance in yeast and transgenic plants. Plant Cell Environ. 29, 1890–1900. doi: 10.1111/j.1365-3040.2006.01565.x, PMID: 16930315

[ref41] Negi J. Moriwaki K. Konishi M. Yokoyama R. Nakano T. Kusumi K.. (2013). A Dof transcription factor, SCAP1, is essential for the development of functional stomata in Arabidopsis. Curr. Biol. 23, 479–484. doi: 10.1016/j.cub.2013.02.001, PMID: 23453954 PMC4039172

[ref42] Negi J. Munemasa S. Song B. Tadakuma R. Fujita M. Azoulay-Shemer T.. (2018). Eukaryotic lipid metabolic pathway is essential for functional chloroplasts and CO_2_ and light responses in Arabidopsis guard cells. Proc. Natl. Acad. Sci. U. S. A. 115, 9038–9043. doi: 10.1073/pnas.1810458115, PMID: 30127035 PMC6130404

[ref43] Nitschke L. Tewari A. Coffin S. L. Xhako E. Pang K. Gennarino V. A.. (2020). miR760 regulates ATXN1 levels via interaction with its 5′ untranslated region. Genes Dev. 34, 1147–1160. doi: 10.1101/gad.339317.120, PMID: 32763910 PMC7462065

[ref44] Obayashi T. Hayashi S. Saeki M. Ohta H. Kinoshita K. (2009). ATTED-II provides coexpressed gene networks for Arabidopsis. Nucleic Acids Res. 37, D987–D991. doi: 10.1093/nar/gkn807, PMID: 18953027 PMC2686564

[ref45] Oh I. S. Park A. R. Bae M. S. Kwon S. J. Kim Y. S. Lee J. E.. (2005). Secretome analysis reveals an Arabidopsis lipase involved in defense against Alternaria brassicicola. Plant Cell 17, 2832–2847. doi: 10.1105/tpc.105.034819, PMID: 16126835 PMC1242276

[ref46] Papanatsiou M. Petersen J. Henderson L. Wang Y. Christie J. M. Blatt M. R. (2019). Optogenetic manipulation of stomatal kinetics improves carbon assimilation, water use, and growth. Science 363, 1456–1459. doi: 10.1126/science.aaw0046, PMID: 30923223

[ref47] Park J. J. Jin P. Yoon J. Yang J. I. Jeong H. J. Ranathunge K.. (2010). Mutation in wilted dwarf and lethal 1 (WDL1) causes abnormal cuticle formation and rapid water loss in rice. Plant Mol. Biol. 74, 91–103. doi: 10.1007/s11103-010-9656-x, PMID: 20593223

[ref48] Rui Y. Xiao C. Yi H. Kandemir B. Wang J. Z. Puri V. M.. (2017). POLYGALACTURONASE INVOLVED IN EXPANSION3 functions in seedling development, rosette growth, and stomatal dynamics in Arabidopsis thaliana. Plant Cell 29, 2413–2432. doi: 10.1105/tpc.17.00568, PMID: 28974550 PMC5774581

[ref49] Su S. H. Bush S. M. Zaman N. Stecker K. Sussman M. R. Krysan P. (2013). Deletion of a tandem gene family in Arabidopsis: increased MEKK2 abundance triggers autoimmunity when the MEKK1-MKK1/2-MPK4 signaling cascade is disrupted. Plant Cell 25, 1895–1910. doi: 10.1105/tpc.113.112102, PMID: 23695980 PMC3694713

[ref50] Su H. G. Zhang X. H. Wang T. T. Wei W. L. Wang Y. X. Chen J.. (2020). Genome-wide identification, evolution, and expression of GDSL-type esterase/lipase gene family in soybean. Front. Plant Sci. 11:726. doi: 10.3389/fpls.2020.00726, PMID: 32670311 PMC7332888

[ref51] Szabados L. Charrier B. Kondorosi A. Debruijn F. J. Ratet P. (1995). New plant promoter and enhancer testing vectors. Mol. Breed. 1, 419–423. doi: 10.1007/BF01248419

[ref52] Takahashi K. Shimada T. Kondo M. Tamai A. Mori M. Nishimura M.. (2010). Ectopic expression of an esterase, which is a candidate for the unidentified plant cutinase, causes cuticular defects in Arabidopsis thaliana. Plant Cell Physiol. 51, 123–131. doi: 10.1093/pcp/pcp173, PMID: 19996150

[ref53] Tang J. Yang X. Xiao C. Li J. Chen Y. Li R.. (2020). GDSL lipase occluded stomatal pore 1 is required for wax biosynthesis and stomatal cuticular ledge formation. New Phytol. 228, 1880–1896. doi: 10.1111/nph.16741, PMID: 32542680

[ref54] Tantikanjana T. Mikkelsen M. D. Hussain M. Halkier B. A. Sundaresan V. (2004). Functional analysis of the tandem-duplicated P450 genes SPS/BUS/CYP79F1 and CYP79F2 in glucosinolate biosynthesis and plant development by Ds transposition-generated double mutants. Plant Physiol. 135, 840–848. doi: 10.1104/pp.104.040113, PMID: 15194821 PMC514119

[ref55] Updegraff E. P. Zhao F. Preuss D. (2009). The extracellular lipase EXL4 is required for efficient hydration of Arabidopsis pollen. Sex. Plant Reprod. 22, 197–204. doi: 10.1007/s00497-009-0104-5, PMID: 20033440

[ref56] Walter M. Chaban C. Schutze K. Batistic O. Weckermann K. Nake C.. (2004). Visualization of protein interactions in living plant cells using bimolecular fluorescence complementation. Plant J. 40, 428–438. doi: 10.1111/j.1365-313X.2004.02219.x, PMID: 15469500

[ref57] Watkins J. L. Li M. McQuinn R. P. Chan K. X. McFarlane H. E. Ermakova M.. (2019). A GDSL esterase/lipase catalyzes the esterification of lutein in bread wheat. Plant Cell 31, 3092–3112. doi: 10.1105/tpc.19.00272, PMID: 31575724 PMC6925002

[ref58] Wu Y. Xun Q. Guo Y. Zhang J. Cheng K. Shi T.. (2016). Genome-wide expression pattern analyses of the Arabidopsis leucine-rich repeat receptor-Like kinases. Mol. Plant 9, 289–300. doi: 10.1016/j.molp.2015.12.011, PMID: 26712505

[ref59] Yang Y. Costa A. Leonhardt N. Siegel R. S. Schroeder J. I. (2008). Isolation of a strong Arabidopsis guard cell promoter and its potential as a research tool. Plant Methods 4:6. doi: 10.1186/1746-4811-4-6, PMID: 18284694 PMC2323621

[ref60] Yeats T. H. Huang W. Chatterjee S. Viart H. M. Clausen M. H. Stark R. E.. (2014). Tomato cutin deficient 1 (CD1) and putative orthologs comprise an ancient family of cutin synthase-like (CUS) proteins that are conserved among land plants. Plant J. 77, 667–675. doi: 10.1111/tpj.12422, PMID: 24372802 PMC3951977

[ref61] Yeats T. H. Martin L. B. Viart H. M. Isaacson T. He Y. Zhao L.. (2012). The identification of cutin synthase: formation of the plant polyester cutin. Nat. Chem. Biol. 8, 609–611. doi: 10.1038/nchembio.960, PMID: 22610035 PMC3434877

[ref62] Yoo S. D. Cho Y. H. Sheen J. (2007). Arabidopsis mesophyll protoplasts: a versatile cell system for transient gene expression analysis. Nat. Protoc. 2, 1565–1572. doi: 10.1038/nprot.2007.199, PMID: 17585298

[ref63] Zhang L. Gao C. Mentink-Vigier F. Tang L. Zhang D. Wang S.. (2019). Arabinosyl deacetylase modulates the Arabinoxylan acetylation profile and secondary wall formation. Plant Cell 31, 1113–1126. doi: 10.1105/tpc.18.00894, PMID: 30886126 PMC6533017

[ref64] Zhang X. Henriques R. Lin S. S. Niu Q. W. Chua N. H. (2006). Agrobacterium-mediated transformation of Arabidopsis thaliana using the floral dip method. Nat. Protoc. 1, 641–646. doi: 10.1038/nprot.2006.97, PMID: 17406292

[ref65] Zhang H. Wang M. Li Y. Yan W. Chang Z. Ni H.. (2020). GDSL esterase/lipases OsGELP34 and OsGELP110/OsGELP115 are essential for rice pollen development. J. Integr. Plant Biol. 62, 1574–1593. doi: 10.1111/jipb.12919, PMID: 32068333

[ref66] Zhang B. Zhang L. Li F. Zhang D. Liu X. Wang H.. (2017a). Control of secondary cell wall patterning involves xylan deacetylation by a GDSL esterase. Nat. Plants 3:17017. doi: 10.1038/nplants.2017.17, PMID: 28260782

[ref67] Zhang H. Zhao Y. Zhou D. X. (2017b). Rice NAD^+^-dependent histone deacetylase OsSRT1 represses glycolysis and regulates the moonlighting function of GAPDH as a transcriptional activator of glycolytic genes. Nucleic Acids Res. 45, 12241–12255. doi: 10.1093/nar/gkx825, PMID: 28981755 PMC5716216

[ref68] Zhao J. Long T. Wang Y. Tong X. Tang J. Li J.. (2020). RMS2 encoding a GDSL lipase mediates lipid homeostasis in anthers to determine Rice male fertility. Plant Physiol. 182, 2047–2064. doi: 10.1104/pp.19.01487, PMID: 32029522 PMC7140947

[ref69] Zhu J. Lou Y. Shi Q. S. Zhang S. Zhou W. T. Yang J.. (2020). Slowing development restores the fertility of thermo-sensitive male-sterile plant lines. Nat. Plants 6, 360–367. doi: 10.1038/s41477-020-0622-6, PMID: 32231254

