# Supplementary material for: Expression Pattern and Functional Analyses of Arabidopsis Guard Cell-Enriched GDSL Lipases
Source: Front Plant Sci. 2021 Sep 21;12:748543. doi: 10.3389/fpls.2021.748543 (PMC8490726; doi:10.3389/fpls.2021.748543)
Supplement: Supplementary file 1 [file Data_Sheet_1.doc]

**Supplementary Figures S1-S9 and Tables S1-S2**

The following Supporting Information is available for this article.

**Figure S1 |** Chromosome distribution of 29 *GGLs*.

**Figure S2 |** *GGLs* showed expressions in trichomes.

**Figure S3 |** Subcellular localization of GGLs in *N. benthamiana* and *Arabidopsis* mesophyll protoplasts.

**Figure S4 |** Phylogenetic relationship and exon-intron structures of 19 GGLs in *Arabidopsis*.

**Figure S5 |** Expression levels of seven *GGLs* in their corresponding T-DNA insertion mutants.

**Figure S6 |** Stomatal density and stomatal morphology of *ggl* mutants.

**Figure S7 |** Expression patterns of 19 *GGLs* under different phytohormone treatments.

**Figure S8 |** Expression patterns of 19 *GGLs* under salty and dehydration conditions.

**Figure S9 |** Expression patterns of some hormone or stress-inducible *GGLs* validated by real-time quantitative PCR.

**Table S1 |** Signal peptides of GGLs predicted by SignalP 4.1.

**Table S2 |** Primers used in this study.


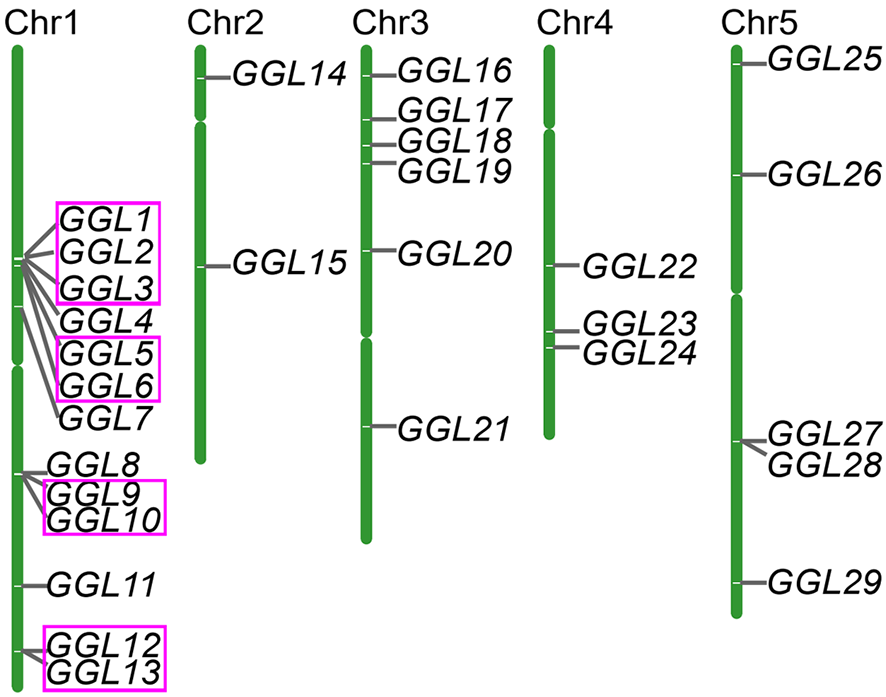


**Figure S1 |** Chromosome distribution of 29 *GGLs*. The diagram showed the distribution of 29 *Arabidopsis* *GGLs* on five chromosomes. Magenta boxes indicated the tandem duplicated *GGLs* analyzed using MCScanX software (Wang et al., 2012).


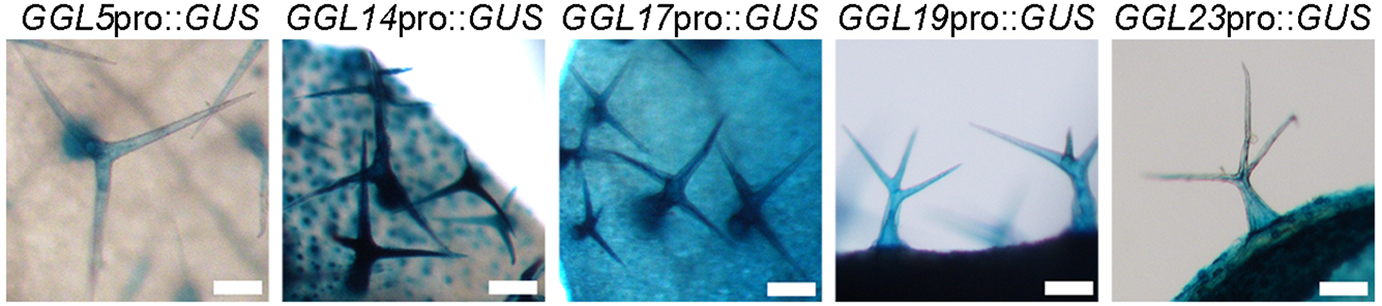


**Figure S2 |** *GGLs* showed expressions in trichomes. Enlarged trichomes in the true leaves of 14-DAG *GGL*pro::*GUS* expressing seedlings (scale bar = 100 μm).

**
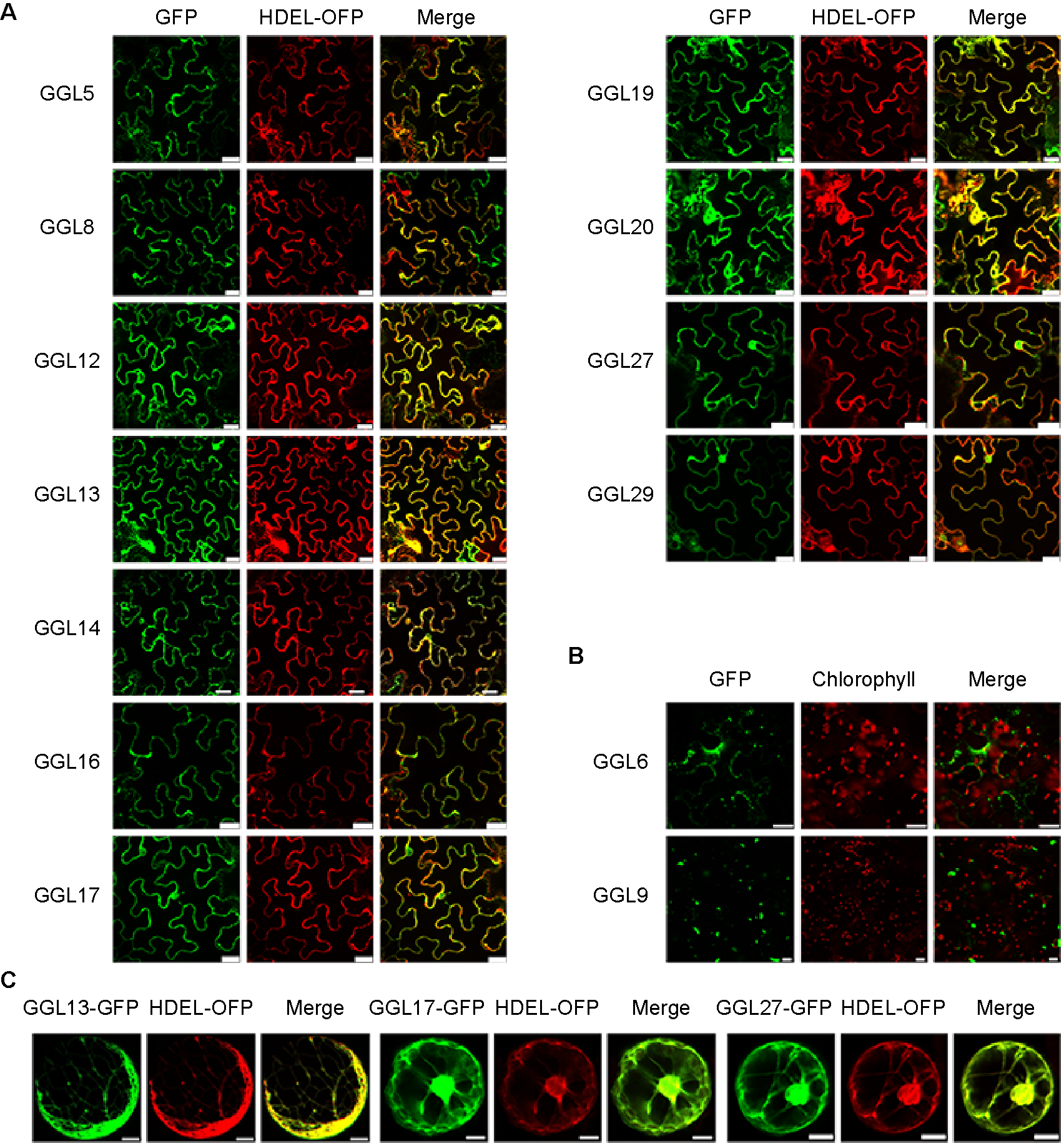
**

**Figure S3 |** Subcellular localization of GGLs in *N. benthamiana* and *Arabidopsis* mesophyll protoplasts. **(A, B)** C-terminal GFP or YFP-tagged GGLs were transiently expressed under the control of the *CaMV 35S* promoter in *N. benthamiana* leaf epidermis. Scale bar = 25 μm. **(C)** Subcellular localization of GGL13, GGL17, and GGL27 in *Arabidopsis* mesophyll protoplasts. Scale bar = 10 μm. HDEL-OFP was coexpressed with GGLs to indicate endoplasmic reticulum localization **(A)** and **(C)**.


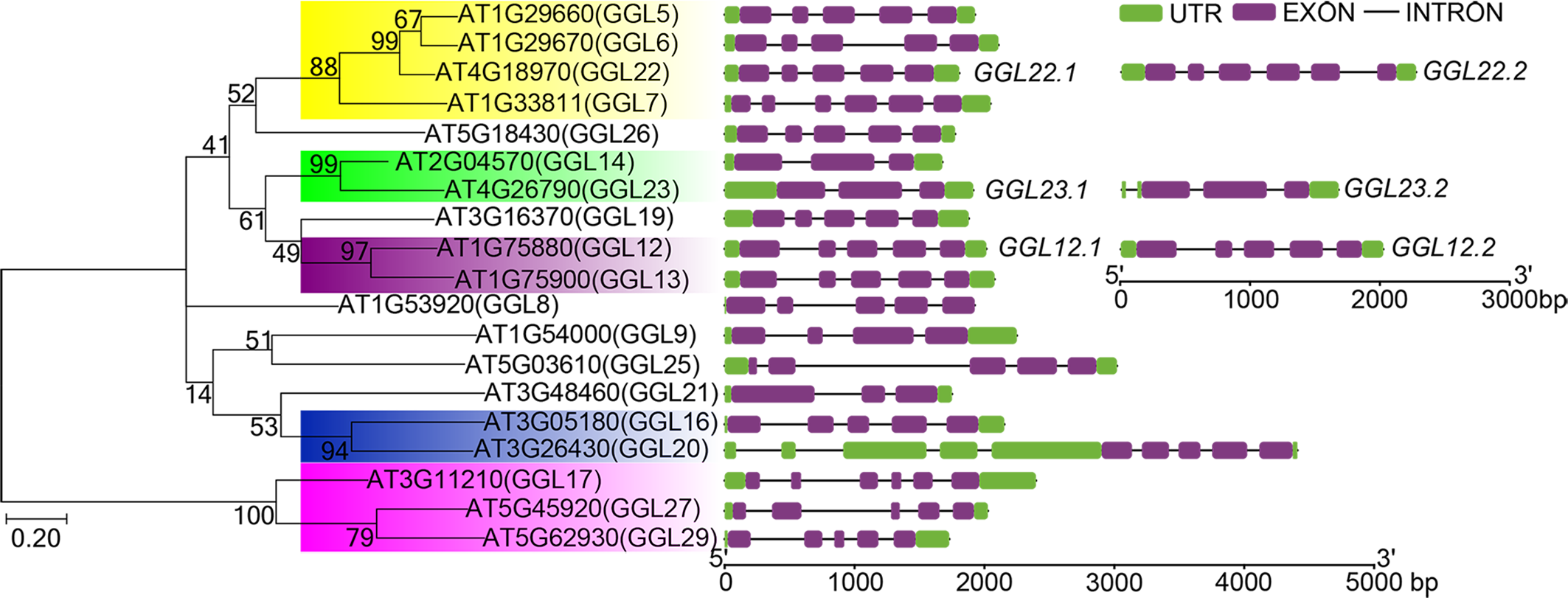


**Figure S4 |** Phylogenetic relationship and exon-intron structures of 19 GGLs in *Arabidopsis*. The phylogenetic relationship of 19 GGLs was analyzed by MEGA 7.0.14. The full-length amino acid sequences of 19 GGLs were used to construct the maximum likelihood tree with 1000 bootstrap replicates, Poisson model, and Complete deletion (the initial tree was automatically made by the Neighbor-Joining method). GGLs with high homology were highlighted with yellow, green, purple, blue, and magenta, respectively. Exon-intron structures were re-drawn by TBtools (Chen et al., 2020). The bottom scales indicated the length of UTRs, exons, and introns of *GGLs*.


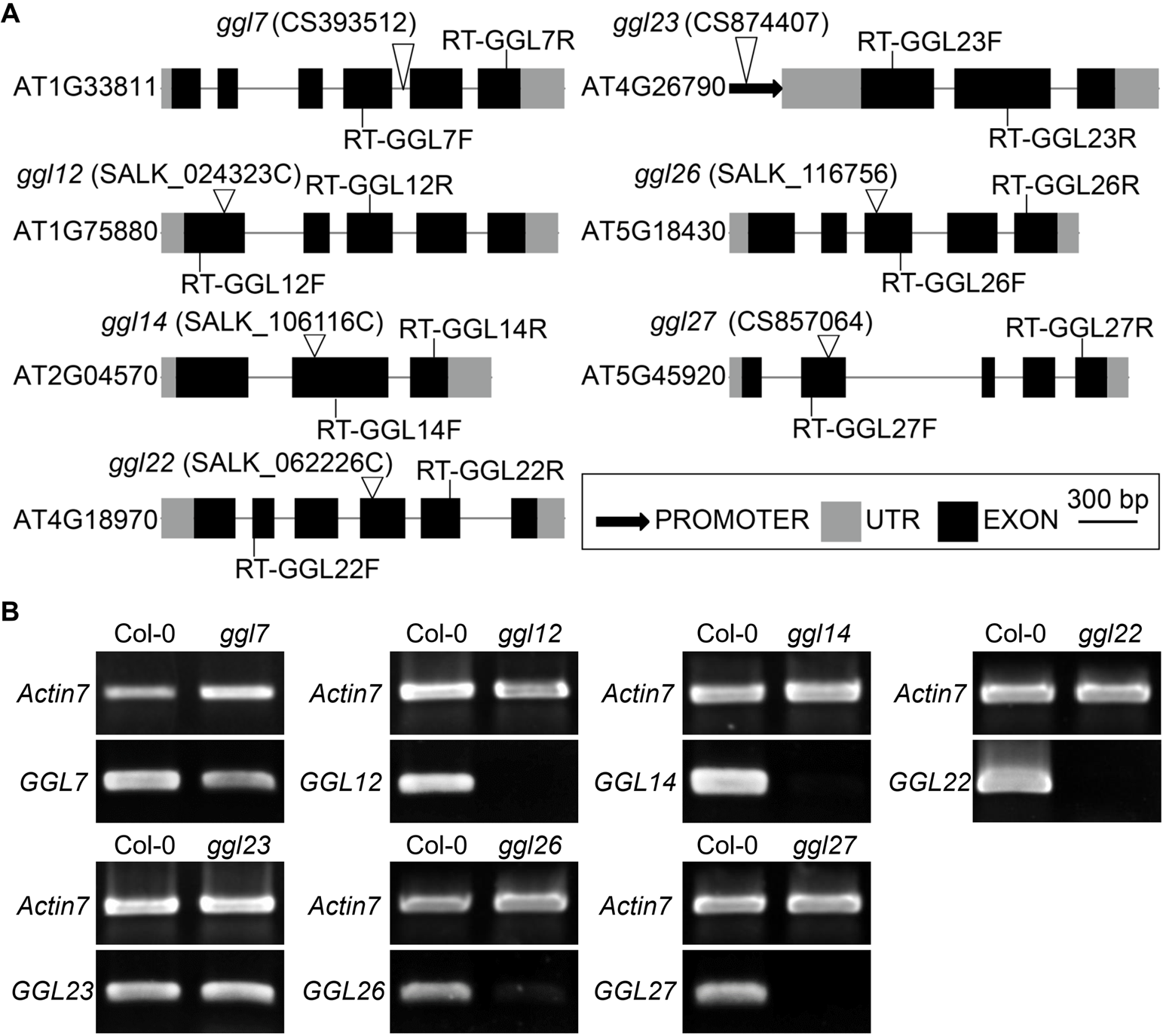


**Figure S5 |** Expression levels of seven *GGLs* in their corresponding T-DNA insertion mutants. **(A)** Schematic diagrams of T-DNA insertion sites in the *ggl* single mutants. **(B)** Expression level analyses of *GGL7*, *GGL12*, *GGL14*, *GGL22*, *GGL23*, *GGL26*, and *GGL27* in their corresponding single mutants by RT-PCR. *Actin7* was used as an internal control.


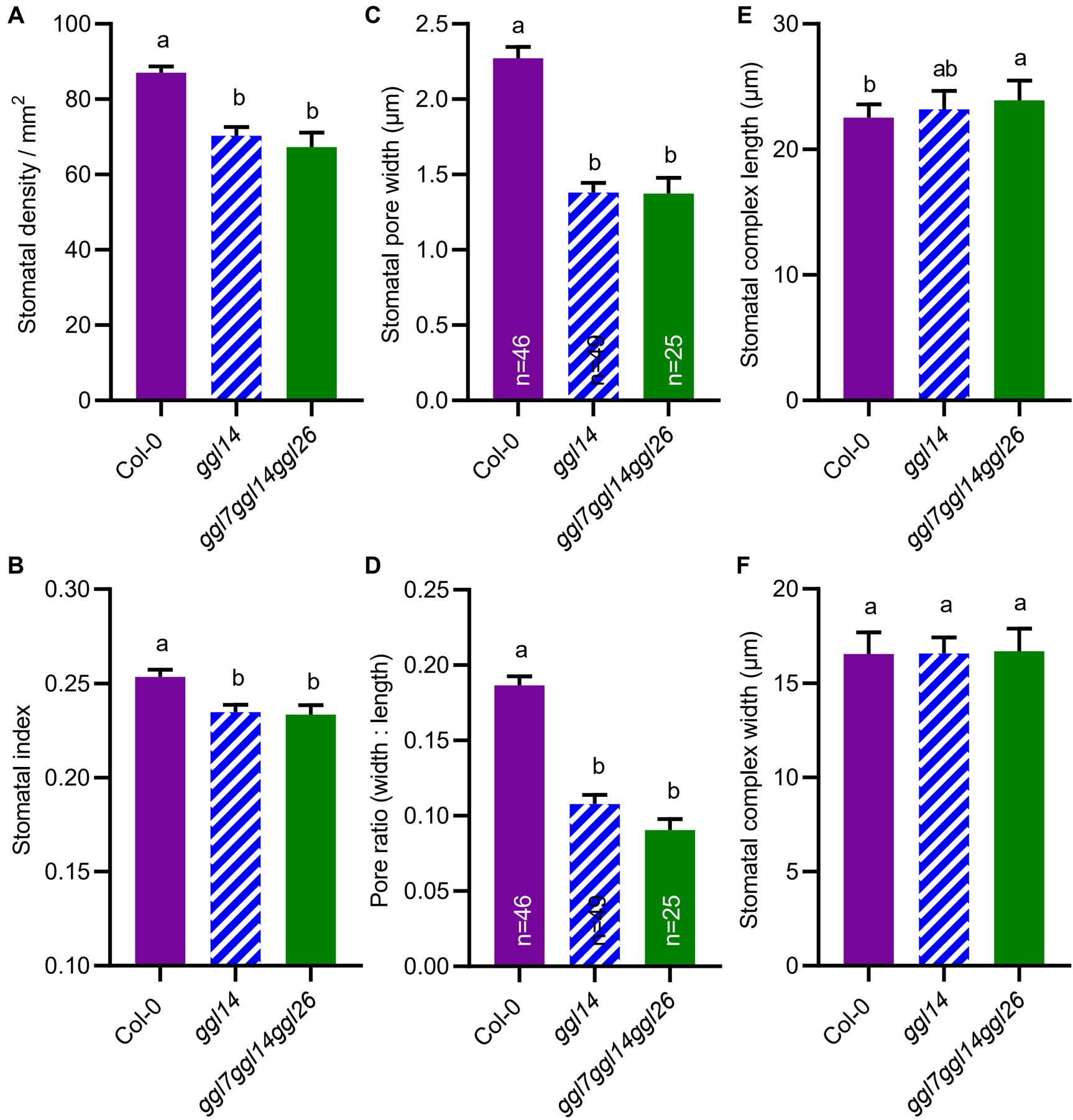


**Figure S6 |** Stomatal density and stomatal morphology of *ggl* mutants. **(A, B)** Stomatal density **(A)** and stomatal index **(B)** in the abaxial leaves of Col-0, *ggl14*, and *ggl7ggl14ggl26*. Values are means ± SE (*n* = 3 independent experiments, each with at least eight leaves per genotype). **(C-F)** Stomatal pore width **(C)**, pore ratio (width : length) **(D)**, stomatal complex length **(E)** and width **(F)** of Col-0, *ggl14*, and *ggl7ggl14ggl26*. Data are means ± SE, *n* = 40 stomata from at least six leaves per genotype **(E)** and **(F)**. Different letters indicate statistically significant differences (*P* < 0.05) by one-way ANOVA and Tukey's test analyses.


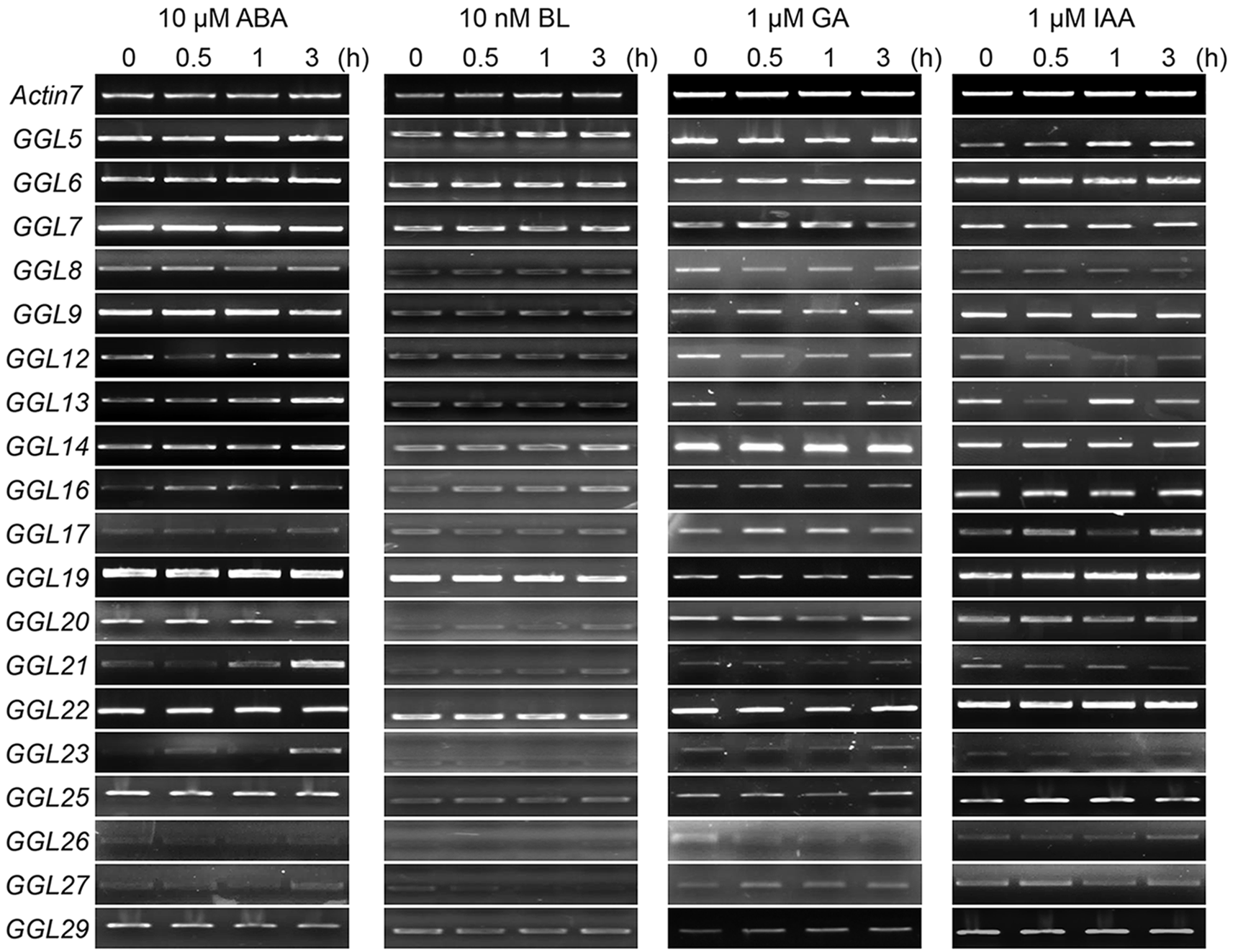


**Figure S7 |** Expression patterns of 19 *GGLs* under different phytohormone treatments. Expression levels of *GGLs* in 7-day-old seedlings treated with 10 μM ABA (Abscisic acid), 10 nM BL (Brassinolide), 1 μM GA (Gibberellin), or 1 μM IAA (Indoleacetic acid) for various times (0, 0.5, 1 and 3 h). *Actin7* was used as an internal control.


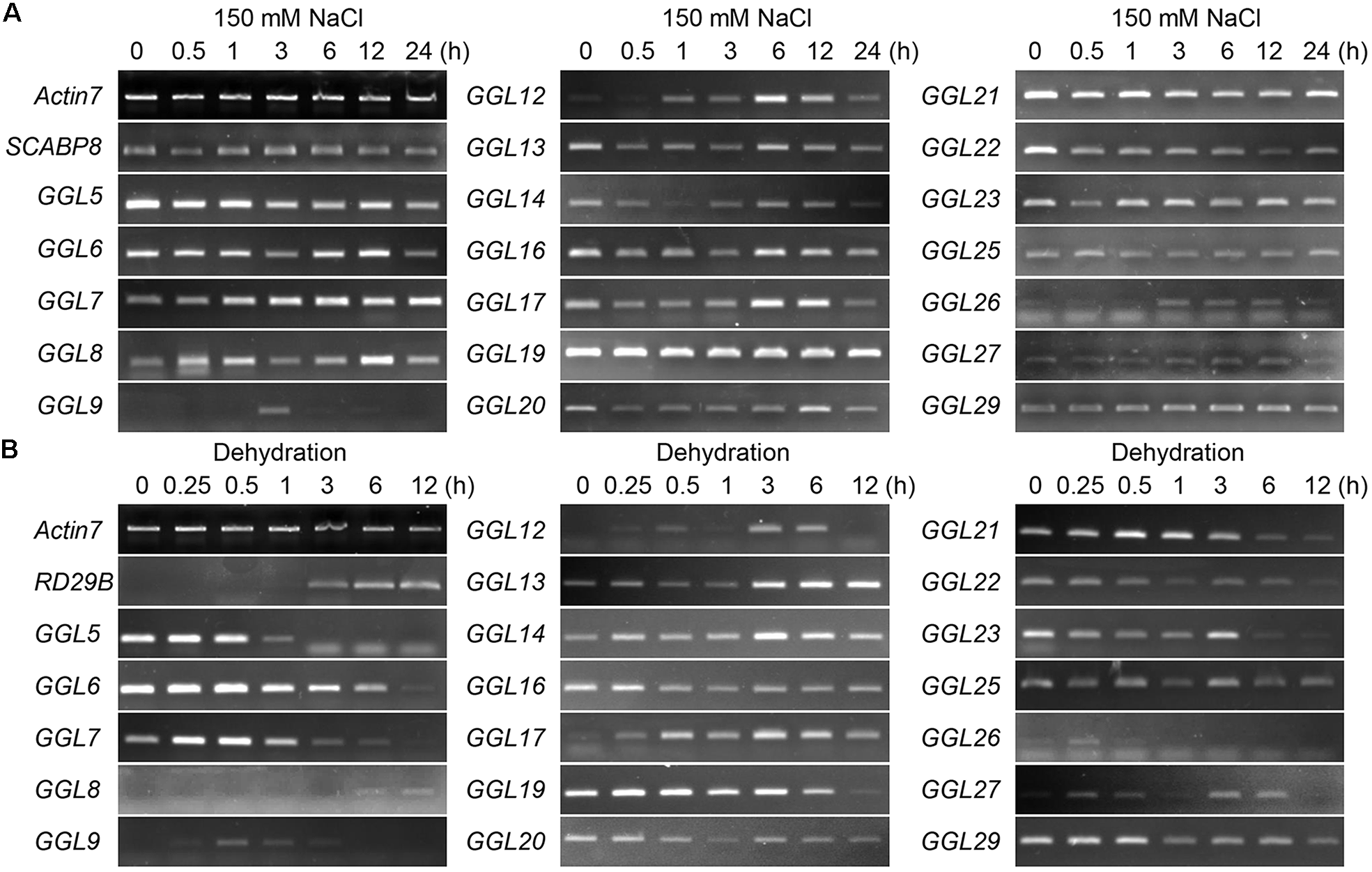


**Figure S8 |** Expression patterns of 19 *GGLs* under salty and dehydration conditions. Expression levels of *GGLs* in the leaves of 4-week-old plants treated with 150 mM NaCl **(A)** or dehydration **(B)** for different times were analyzed. *SCABP8* and *RD29B* were used as positive controls for 150 mM NaCl or dehydration treatments, respectively. *Actin7* was used as an internal control.


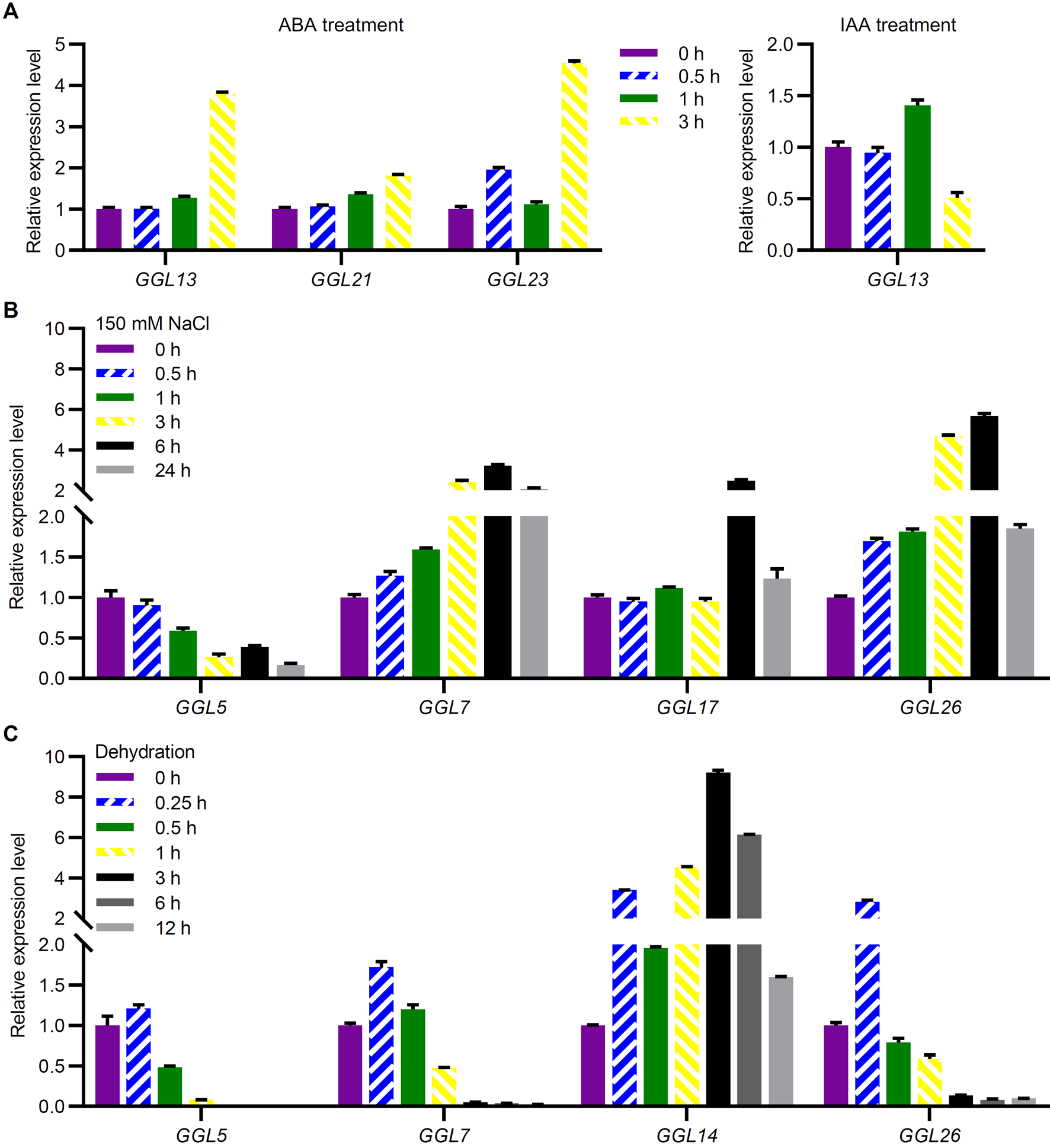


**Figure S9 |** Expression patterns of some hormone or stress-inducible *GGLs* validated by real-time quantitative PCR. **(A)** Expression profiles of *GGL13*, *GGL21*, and *GGL23* under ABA (10 μM) or IAA (1 μM) treatment. **(B)** Expression patterns of *GGL5*, *GGL7*, *GGL17*, and *GGL26* under salty conditions. **(C)** Expression patterns of *GGL5*, *GGL7*, *GGL14*, and *GGL26* under dehydration conditions. Values are means ± SE (*n* = 3).

**Table S1 |** **Signal peptides of GGLs predicted by SignalP 4.1.**

| Gene No. | sequence ID | source | feature | start | end | score | N/A |
| --- | --- | --- | --- | --- | --- | --- | --- |
| GGL5 | AT1G29660 | SignalP-4.1 | SIGNAL | 1 | 26 | 0.848 | YES |
| GGL6 | AT1G29670 | SignalP-4.1 | SIGNAL | 1 | 24 | 0.914 | YES |
| GGL7 | AT1G33811 | SignalP-4.1 | SIGNAL | 1 | 24 | 0.723 | YES |
| GGL8 | AT1G53920 | SignalP-4.1 | SIGNAL |  |  | 0.312 | NO |
| GGL9 | AT1G54000 | SignalP-4.1 | SIGNAL | 1 | 29 | 0.767 | YES |
| GGL12 | AT1G75880.1 | SignalP-4.1 | SIGNAL |  |  | 0.429 | NO |
| GGL13 | AT1G75900 | SignalP-4.1 | SIGNAL | 1 | 32 | 0.733 | YES |
| GGL14 | AT2G04570 | SignalP-4.1 | SIGNAL | 1 | 23 | 0.89 | YES |
| GGL16 | AT3G05180 | SignalP-4.1 | SIGNAL | 1 | 27 | 0.752 | YES |
| GGL17 | AT3G11210 | SignalP-4.1 | SIGNAL |  |  | 0.219 | NO |
| GGL19 | AT3G16370 | SignalP-4.1 | SIGNAL | 1 | 25 | 0.852 | YES |
| GGL20 | AT3G26430 | SignalP-4.1 | SIGNAL | 1 | 23 | 0.616 | YES |
| GGL21 | AT3G48460 | SignalP-4.1 | SIGNAL | 1 | 26 | 0.763 | YES |
| GGL22 | AT4G18970.1 | SignalP-4.1 | SIGNAL | 1 | 22 | 0.782 | YES |
| GGL23 | AT4G26790.1 | SignalP-4.1 | SIGNAL | 1 | 25 | 0.76 | YES |
| GGL25 | AT5G03610 | SignalP-4.1 | SIGNAL | 1 | 26 | 0.783 | YES |
| GGL26 | AT5G18430 | SignalP-4.1 | SIGNAL | 1 | 19 | 0.829 | YES |
| GGL27 | AT5G45920 | SignalP-4.1 | SIGNAL |  |  | 0.125 | NO |
| GGL29 | AT5G62930 | SignalP-4.1 | SIGNAL |  |  | 0.141 | NO |

**Table S2 | Primers used in this study.**

| Primer name | Primer sequence | Application of primer |
| --- | --- | --- |
| GGL5Pro-GUS-F | CG*GGATCC*GGACCCGATCCAGTTAAAGTGCT | Expression pattern analyses |
| GGL5Pro-GUS-R | GG*GGTACC*AGTGTAGGAGTGGTGGTCGTTGT |  |
| GGL6Pro-GUS-F1 | *GGGGACAAGTTTGTACAAAAAAGCAGGCT*AAGGACAGAGAATAGGAGAG |  |
| GGL6Pro-GUS-R1 | *GGGGACCACTTTGTACAAGAAAGCTGGGT*TTCAAGACCAATATCTTTTT |  |
| GGL7Pro-GUS-F | ACGGATCCGACCTACTTCCCCTTCGACT |  |
| GGL7Pro-GUS-F | ACGGTACCTAAGTTATAATTTAGTGAAA |  |
| GGL8Pro-GUS-F1 | *GGGGACAAGTTTGTACAAAAAAGCAGGCT*GATGATTTCGACGTCGGTGATGT |  |
| GGL8Pro-GUS-R1 | *GGGGACCACTTTGTACAAGAAAGCTGGGT*TTCTAAAGACTCGTGATGTGTGTGT |  |
| GGL9Pro-GUS-F1 | *GGGGACAAGTTTGTACAAAAAAGCAGGCT*TTTTGGAGCGGACCCAAATG |  |
| GGL9Pro-GUS-R1 | *GGGGACCACTTTGTACAAGAAAGCTGGGT*GCTTTTGTGCCTTAGCTCGTAG |  |
| GGL12Pro-GUS-F | CG*GGATCC*CTTAAACAAGACGGTTGG |  |
| GGL12Pro-GUS-R | TCC*CCCGGG*AGTTGCTAATTACTATGACG |  |
| GGL13Pro-GUS-F1 | *GGGGACAAGTTTGTACAAAAAAGCAGGCT*CATAATTATGTTAGATAG |  |
| GGL13Pro-GUS-R1 | *GGGGACCACTTTGTACAAGAAAGCTGGGT*GTTGCTTTAATGTAATGTAT |  |
| GGL14Pro-GUS-F | ACGTCGACGTTATCGCCGGAAACTTCGC |  |
| GGL14Pro-GUS-R | ACGGTACCAGTGTTTGTTTTTGTGGTTA |  |
| GGL16Pro-GUS-F | CG*GGATCC*AGGGTGTACCTTCTCAAA |  |
| GGL16Pro-GUS-R | GG*GGTACC*GGCTCAAAACAATATAATTTC |  |
| GGL17Pro-GUS-F | CG*GGATCC*TGTTACTTTCCCGCCTTCC |  |
| GGL17Pro-GUS-R | GG*GGTACC*TGTCTCCTTCCTAACCTCTC |  |
| GGL19Pro-GUS-F | CG*GGATCC*TCCGCTGAACCTTGAACT |  |
| GGL19Pro-GUS-R | GG*GGTACC*GTTTTTGCTTTGGTATGTGA |  |
| GGL20Pro-GUS-F | CG*GGATCC*TGTGGCAAGCCTGGACAT |  |
| GGL20Pro-GUS-R | GG*GGTACC*TTTCTCCTTTTATCTTCTTC |  |
| GGL21Pro-GUS-F | CG*GGATCC*GTCACTAATAACCCAATC |  |
| GGL21Pro-GUS-R | GG*GGTACC*TGTGATAAGGAAGTGAGA |  |
| GGL22Pro-GUS-F1 | *GGGGACAAGTTTGTACAAAAAAGCAGGCT*CTTGTTCGCTAGGATGAT |  |
| GGL22Pro-GUS-R1 | *GGGGACCACTTTGTACAAGAAAGCTGGGT*CTCTACCCTTATCCGGAAAG |  |
| GGL23Pro-GUS-F | CG*GGATCC*TAGACATCTGAGCTGGAA |  |
| GGL23Pro-GUS-R | GG*GGTACC*GCTTTTTACACAAGTTTTTTC |  |
| GGL25Pro-GUS-F | CG*GGATCC*CAAGTGACGGTGGGAAGT |  |
| GGL25Pro-GUS-R | GG*GGTACC*TTTCAAGATTCAGAGCAAAG |  |
| GGL26Pro-GUS-F | CG*GGATCC*GACCCATAAGGTTACTGT |  |
| GGL26Pro-GUS-R | GG*GGTACC*AAAAGCGTTTTGTTTAATG |  |
| GGL27Pro-GUS-F | CG*GAATTC*CTTCAGATTATACCAAACCCAA |  |
| GGL27Pro-GUS-R | CG*GGATCC*AAAATCTGAGAGATAAGATAG |  |
| GGL29Pro-GUS-F1 | *GGGGACAAGTTTGTACAAAAAAGCAGGCT*ATTATGCAATATCCACCAC |  |
| GGL29Pro-GUS-R1 | *GGGGACCACTTTGTACAAGAAAGCTGGGT*CTCGCTGTCTCCCTCCGG |  |
| GGL5-GFP-F | *GGGGACAAGTTTGTACAAAAAAGCAGGCTTA*ATGGAGAGTTACTTGAGG | Subcellular localization analyses |
| GGL5-GFP-R | *GGGGACCACTTTGTACAAGAAAGCTGGGTA*AAGCTGTGCTAATTGCGAG |  |
| GGL6-GFP-F | *GGGGACAAGTTTGTACAAAAAAGCAGGCTTA*ATGGAGAGTTACTTAACG |  |
| GGL6-GFP-R | *GGGGACCACTTTGTACAAGAAAGCTGGGTA*AAGCTGTGCCAGCCTTG |  |
| GGL8-GFP-F | *GGGGACAAGTTTGTACAAAAAAGCAGGCTTA*ATGAGAGAGTCAACTTTAATG |  |
| GGL8-GFP-R | *GGGGACCACTTTGTACAAGAAAGCTGGGTA*AGGAATCTGAAATAAATTATTG |  |
| GGL9-GFP-F | *GGGGACAAGTTTGTACAAAAAAGCAGGCTTA*ATGATGGCAAACAACTGT |  |
| GGL9-GFP-R | *GGGGACCACTTTGTACAAGAAAGCTGGGTA*GTAATACTCGTAACCGCG |  |
| GGL12-GFP-F | *GGGGACAAGTTTGTACAAAAAAGCAGGCTTA*ATGGAACGATGTCGTTCT |  |
| GGL12-GFP-R | *GGGGACCACTTTGTACAAGAAAGCTGGGTA*GAAGAATCTGTTGAGGTATC |  |
| GGL13-GFP-F | *GGGGACAAGTTTGTACAAAAAAGCAGGCTTA*GCCTCTGTTTGTCCTTGTTATGT |  |
| GGL13-GFP-R | *GGGGACCACTTTGTACAAGAAAGCTGGGTA*CCAAAACACATGAGTAGACACG |  |
| GGL14-GFP-F | *GGGGACAAGTTTGTACAAAAAAGCAGGCT*ACAAACACTATGGGACACC |  |
| GGL14-GFP-R | *GGGGACCACTTTGTACAAGAAAGCTGGGTA*GAGGAAGTGAGGGAATGT |  |
| GGL16-GFP-F | *GGGGACAAGTTTGTACAAAAAAGCAGGCTTA*ATGGAAACTCTTTTCCAC |  |
| GGL16-GFP-R | *GGGGACCACTTTGTACAAGAAAGCTGGGTA*AAGATTCAAAGATGACGC |  |
| GGL17-GFP-F | *GGGGACAAGTTTGTACAAAAAAGCAGGCTTA*ATGGTTGGACCCGCGCGG |  |
| GGL17-GFP-R | *GGGGACCACTTTGTACAAGAAAGCTGGGTA*GTCCCATTGTTCTTCCCAG |  |
| GGL19-GFP-F | *GGGGACAAGTTTGTACAAAAAAGCAGGCTTA*ATGGATCGTTGCACGTCG |  |
| GGL19-GFP-R | *GGGGACCACTTTGTACAAGAAAGCTGGGTA*ACCGAGGAGAGAAAAGCC |  |
| GGL20-GFP-F | *GGGGACAAGTTTGTACAAAAAAGCAGGCTTA*ATGGAAACTAATCTCTTG |  |
| GGL20-GFP-R | *GGGGACCACTTTGTACAAGAAAGCTGGGTA*TCTGGTGCAGGCAGACTTC |  |
| GGL27-GFP-F | *GGGGACAAGTTTGTACAAAAAAGCAGGCTTA*ATGATGAGACGGAAGATT |  |
| GGL27-GFP-R | *GGGGACCACTTTGTACAAGAAAGCTGGGTA*AAACTCATCAAAGGATTTG |  |
| GGL29-GFP-F | *GGGGACAAGTTTGTACAAAAAAGCAGGCTTA*ATGAGGCCGGAGATAGTT |  |
| GGL29-GFP-R | *GGGGACCACTTTGTACAAGAAAGCTGGGTA*TAAGCAACGCTCTTCAAAAG |  |
| RT-GGL5F | AGCAGACAGTACACTCCCGA | RT-PCR analyses |
| RT-GGL5R | TCATCTCGGTTCAAGCACGG |  |
| RT-GGL6F | TACGGTATCGATTTCGGCGG |  |
| RT-GGL6R | TTGCTGAGTTGATGCGGTCT |  |
| RT-GGL7F | ACTCGGGAATGGGAAGTAAC |  |
| RT-GGL7R | TAGATTTCGCCAGCAGTATGT |  |
| RT-GGL8F | TCCGACGGTCGTCTCATC |  |
| RT-GGL8R | ACAACCCAAGTCAGGCACA |  |
| RT-GGL9F | TCGCTCAAGGTTTCTGGC |  |
| RT-GGL9R | TTTCGTTCAACATCGGTCC |  |
| RT-GGL12F | TTTGGTGTATTTTCGTTCTCG |  |
| RT-GGL12R | GCGACGGTGTAGTGTAGCC |  |
| RT-GGL13F | AGTTGGGGAAGCAAGAAAAG |  |
| RT-GGL13R | AGACACGTCGGGACATACAG |  |
| RT-GGL14F | AGTTGCATGGACTTGGTGC |  |
| RT-GGL14R | AGGAAGTGAGGGAATGTGCT |  |
| RT-GGL16F | TAGCCCCTTTGGTTTTGG |  |
| RT-GGL16R | TGATTCAGGATGAGGTCGG |  |
| RT-GGL17F | TTCTTTCCGAGGTCTACGC |  |
| RT-GGL17R | TGTGCTGACAAATGAATCCC |  |
| RT-GGL19F | ACAACAACTATCTTCCGACCC |  |
| RT-GGL19R | TGCTCCAACCGCATACACT |  |
| RT-GGL20F | AACGCAACCATCGCTCAG |  |
| RT-GGL20R | TTCCCACCATGTCCACAAC |  |
| RT-GGL21F | GATTCTTTCACCGACACCG |  |
| RT-GGL21R | GCCAACGACATTGCTAACG |  |
| RT-GGL22F | CTGAGCTTCTTGGTTTCGATG |  |
| RT-GGL22R | ACTACCGATGACAACATTCGC |  |
| RT-GGL23F | GCCGTGTTATCTGTAATGCC |  |
| RT-GGL23R | CCGAATGCTTCAGGATGG |  |
| RT-GGL25F | TTCCAGAACATTCTCACCACC |  |
| RT-GGL25R | AGTTATACTCGCGGCTCACA |  |
| RT-GGL26F | AGTCGCTTGATTGGGAAACC |  |
| RT-GGL26R | CCATTGTACGGCCCTTGTC |  |
| RT-GGL27F | AACACGAGGTGGGCACTG |  |
| RT-GGL27R | CGACCCGACTCAAATGTAAC |  |
| RT-GGL29F | ACGGCGGCTACAACACC |  |
| RT-GGL29R | AACTACCCCATTGCCTTCA |  |
| RTSCABP8F | AGCTTAGACTGGCGCTGTTC |  |
| RTSCABP8R | TTCCTCGTTCTGCTCTTGCG |  |
| Actin7-F | GGCCGATGGTGAGGATATTCAGCCACTTG |  |
| Actin7-R | TCGATGGACCTGACTCATCGTACTCACTC |  |
| RD29B-BDL-L | ATGGAGTCACAGTTGACACG |  |
| RD29B-BDL-R | CAGGCTCAATGGGTTTGGTG |  |
| EFα-F | AATGGTGACGCTGGTATG | Real-time PCR analyses |
| EFα-R | CTTCTTGTCCACGCTCTT |  |
| qGGL5F | TCCGACGTTTATCCGATTGAT |  |
| qGGL5R | ACACTTTGGGTACACACATAACATC |  |
| qGGL7F | TCATCCGACGGAGACTGCTA |  |
| qGGL7R | TGCATTCATATGGGAGATCAAGA |  |
| qGGL13F | TGTCCCGACGTGTCTACTCA |  |
| qGGL13R | TCAATCATTATGCCGTGAAAATAGT |  |
| qGGL14F | CGAGATGGGATATGGTTGTCA |  |
| qGGL14R | GGGAATGTGCTGTTCATGAGA |  |
| qGGL17F | AGCAGATGGCAAACAGACAGT |  |
| qGGL17R | CAGTGAATTATGTGAAGATAGGGTC |  |
| qGGL21F | TTCCAAACGTGTGGGACGG |  |
| qGGL21R | TAGGACGAGTGAAAGTGCCG |  |
| qGGL23F | GAAGACAAACGCCATCGTAGC |  |
| qGGL23R | TAGTGCTTGTGGTGTAGCAAT |  |
| qGGL26F | GCAAACCGCATGATAGTCCG |  |
| qGGL26R | TTCCCACTTCAACGTGTGTG |  |
